# Spatiotemporally resolved colorectal oncogenesis in mini-colons ex vivo

**DOI:** 10.1038/s41586-024-07330-2

**Published:** 2024-04-24

**Authors:** L. Francisco Lorenzo-Martín, Tania Hübscher, Amber D. Bowler, Nicolas Broguiere, Jakob Langer, Lucie Tillard, Mikhail Nikolaev, Freddy Radtke, Matthias P. Lutolf

**Affiliations:** 1 Laboratory of Stem Cell Bioengineering, Institute of Bioengineering, School of Life Sciences and School of Engineering, École Polytechnique Fédérale de Lausanne (EPFL), Lausanne, Switzerland; 2 Swiss Institute for Experimental Cancer Research (ISREC), School of Life Sciences, Ecole Polytechnique Fédérale de Lausanne (EPFL), Lausanne, Switzerland; 3 Swiss Cancer Center Leman (SCCL), Lausanne, Switzerland; 4 Institute of Human Biology (IHB), Roche Pharma Research and Early Development, Roche Innovation Center Basel, Basel, Switzerland

**Keywords:** Cancer models, Tissue engineering, Gastrointestinal cancer, Lab-on-a-chip

## Abstract

Three-dimensional organoid culture technologies have revolutionized cancer research by allowing for more realistic and scalable reproductions of both tumour and microenvironmental structures^1–3^. This has enabled better modelling of low-complexity cancer cell behaviours that occur over relatively short periods of time^4^. However, available organoid systems do not capture the intricate evolutionary process of cancer development in terms of tissue architecture, cell diversity, homeostasis and lifespan. As a consequence, oncogenesis and tumour formation studies are not possible in vitro and instead require the extensive use of animal models, which provide limited spatiotemporal resolution of cellular dynamics and come at a considerable cost in terms of resources and animal lives. Here we developed topobiologically complex mini-colons that are able to undergo tumorigenesis ex vivo by integrating microfabrication, optogenetic and tissue engineering approaches. With this system, tumorigenic transformation can be spatiotemporally controlled by directing oncogenic activation through blue-light exposure, and emergent colon tumours can be tracked in real-time at the single-cell resolution for several weeks without breaking the culture. These induced mini-colons display rich intratumoural and intertumoural diversity and recapitulate key pathophysiological hallmarks displayed by colorectal tumours in vivo. By fine-tuning cell-intrinsic and cell-extrinsic parameters, mini-colons can be used to identify tumorigenic determinants and pharmacological opportunities. As a whole, our study paves the way for cancer initiation research outside living organisms.

## Abstract (web summary)

Topobiologically complex mini-colons—which enable the faithful in vitro recapitulation of colorectal cancer tumorigenesis and its environmental determinants—offer the possibility to reduce animal use in a wide range of experimental applications.

## Main

Cancer arises through the accumulation of genetic lesions that confer unrestrained cell growth potential. Over the past 70 years, both two-dimensional (2D) and three-dimensional (3D) in vitro culture models have been developed to make simplified, animal-free versions of cancers readily available for research^4^. These models successfully portray and dissect a wide range of relatively simple cancer cell behaviours, such as proliferation, motility, invasiveness, survival, cell–cell and cell–stroma interactions, and drug responses, among others^1,2,4^. However, modelling more complex processes that involve multiple cell (sub)types and tissue-level organization remains a challenge, as is the case for cancer initiation.

The cellular transition from healthy to cancerous is an intricate evolutionary process that is still largely obscure due to the insufficient topobiological complexity of the available in vitro cell culture systems, which precludes de novo tumour generation and the establishment of pathophysiologically relevant tumorigenic models^5,6^. Even the current gold-standard organoid-based 3D models, which are often postulated as a bridge between in vitro and in vivo^1,3,7^, are too simplified for modelling cancer development ex vivo. This is mostly due to (1) their closed cystic structure instead of an in vivo-like apically open architecture^8^; (2) their short lifespan that requires breaking up the culture every few days for passaging^9^; (3) their lack of topobiological stability and consistency owing to their stochastic growth in 3D matrices^8^; and (4) their inability to generate hybrid tissues composed of healthy and cancer cells in a balanced and integrated manner^10^. Various next-generation approaches such as bioprinting and microfabrication technologies have been recently implemented to partially address some of these issues^11,12^; however, none have been able to fully recreate intratumour and intertumour complexity. Consequently, cancer research is still inevitably bound to animal experimentation, which provides a pathophysiologically relevant setting, but forbids high-resolution and real-time analyses of cellular dynamics during oncogenesis. Moreover, these models are economically and ethically costly. Thus, while there is the widespread consensus that animal use in research should be reduced, replaced and refined (the 3 Rs^13^), this commitment is severely hindered by the insufficient physiological complexity displayed by classical in vitro systems.

Here we postulated that a 3D system able to solve the existing limitations of in vitro cultures could be engineered by leveraging scaffold-guided organoid morphogenesis and optogenetics. Specifically, we developed miniature colon tissues in which cells could (1) be cultured for long durations (several weeks) without the need for breaking the culture through passaging; (2) reproduce the stem-differentiated cell patterning axis in a stable and anatomically relevant topology; (3) be easily mutated and tracked in a spatiotemporally controlled manner; and (4) create a biomechanically dynamic system that allows for tumour emergence while preserving the integrity of the surrounding healthy tissue. These features permit the development of biologically complex tumours ex vivo, bridging the gap between in vitro and in vivo models by providing a high-resolution system that can be used to dissect the molecular factors orchestrating cancer initiation.

## Spatiotemporally regulated tumorigenesis

**Extended Data Fig. 1:**
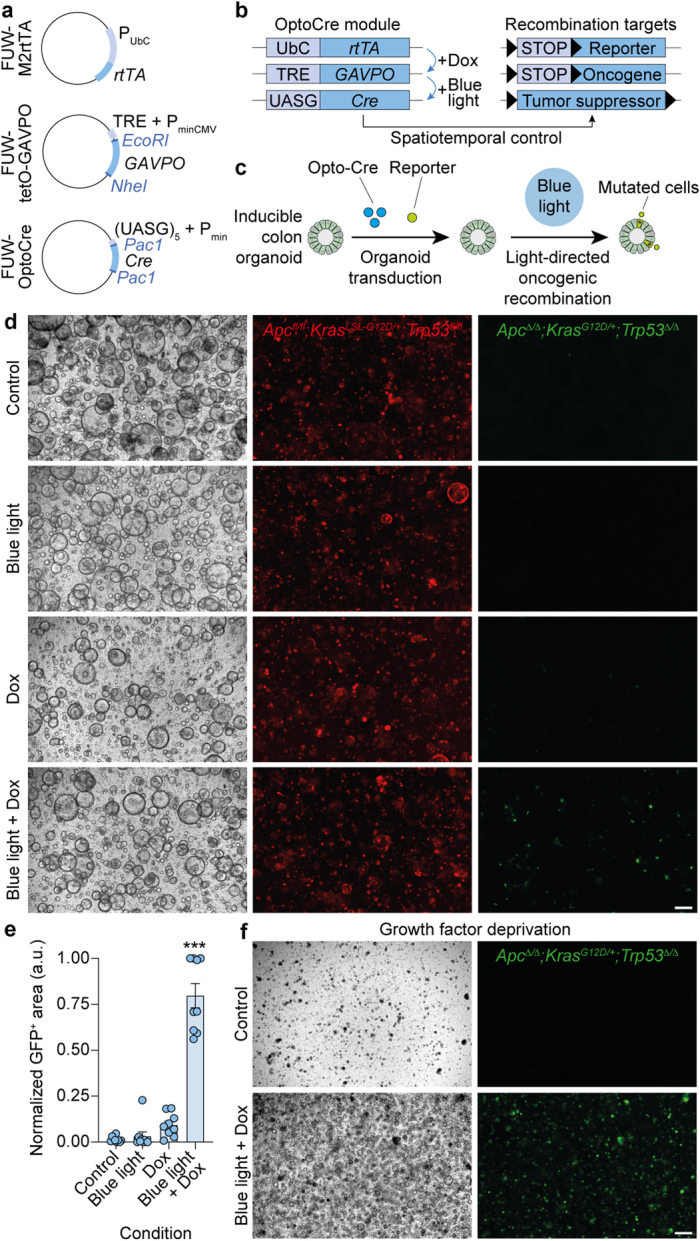
Generation of blue–light-inducible AKP colon organoids. **a**, Schematic of the plasmids comprising the OptoCre module. The promoter, gene, and restriction sites used to generate the plasmids are indicated. **b**, Schematic of the integration of the different genetic elements that allow spatiotemporal control of oncogenic recombination in colon organoids. **c**, Schematic of the experimental workflow used to test and optimize the OptoCre system. **d**, Brightfield and fluorescence images of OptoCre-carrying inducible colon organoids exposed to the indicated conditions. Red and green signals correspond to healthy and mutated cells, respectively. Images were taken 48 h after induction. Scale bar, 200 μm. **e**, Recombination efficiency in inducible colon organoid exposed to the conditions indicated in panel d. ***P = 0.0001 (Kruskal-Wallis and Dunn’s multiple comparisons test; *n* = 9 cultures for each condition). Each point represents one well of organoids. Data represent mean ± SEM. **f**, Brightfield and fluorescence images of inducible colon organoids exposed to control (top) and activation (bottom) conditions, dissociated into single cells and replated in the absence of growth factors (BMGF medium). Green signal corresponds to mutated cells. Images were taken 24 h after replating. Scale bar, 200 μm. Source Data

We focused on colorectal cancer (CRC) as it is one of the most prominent cancer types worldwide and its malignant transformation can be readily engineered genetically^14,15^. To first achieve spatiotemporal control of oncogenic DNA recombination, we developed a doxycycline-sensitive blue-light-regulated Cre system (hereafter, OptoCre), which we then introduced into inducible *Apc*^*fl/fl*^*Kras*^*LSL-G12D/+*^*Trp53*^*fl/fl*^ (AKP) healthy colon organoids (Extended Data Fig. 1a–c). A fluorescent Cre reporter was also incorporated to track cells that undergo oncogenic recombination (Extended Data Fig. 1b,c). We initially tested the system in conventional organoid cultures, in which OptoCre efficiently induced recombination in the presence of blue light and doxycycline (Extended Data Fig. 1d,e). Dosage optimization prevented unwanted activation by coupling high efficiency with low leakiness (~1.6%) (Extended Data Fig. 1d,e). To confirm successful oncogenic transformation, we removed growth factors (EGF, noggin, R-spondin, WNT3A) from the organoid medium and observed that only cells with an activated OptoCre were able to grow, a well-known hallmark of mutated AKP colon organoids^16^ (Extended Data Fig. 1f). The presence of the expected mutations at the *Apc, Kras* and *Trp53* loci was confirmed by PCR and exome sequencing (see below; Extended Data Fig. 3f,g).

**Extended Data Fig. 2:**
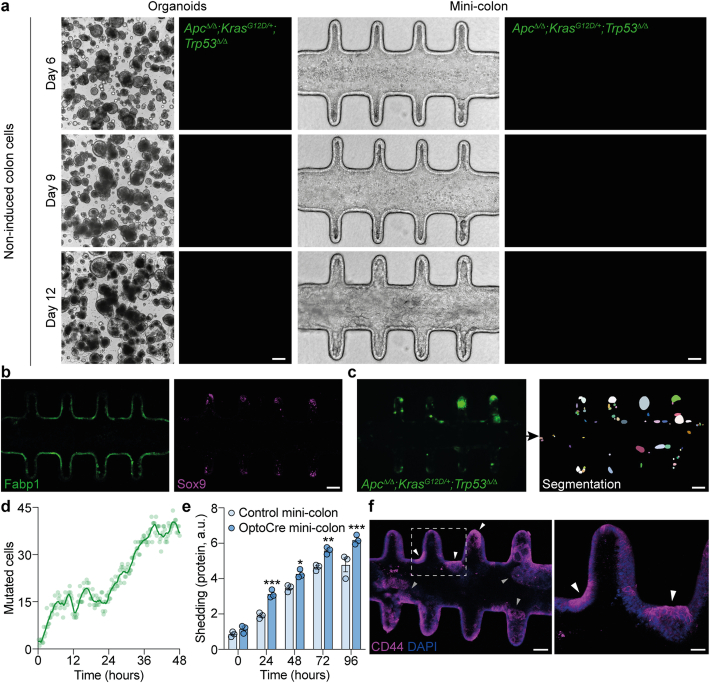
Oncogenic mutations induce neoplastic growth in mini-colons. **a**, Time-course brightfield and fluorescence images of non-induced healthy colon cells grown as conventional organoids and mini-colons. Absence of fluorescence signal indicates absence of oncogenic recombination. Scale bars, 200 μm (organoids) and 75 μm (mini-colons). **b**, Immunofluorescence images showing the expression of *Fabp1* (left, green), and *Sox9* (right, magenta) in healthy mini-colons cultured for 7 days. Scale bar, 100 μm. **c**, Fluorescence image (left) showing the presence of mutated cells 36 h after the blue–light-mediated induction of a mini-colon. The segmentation of each mutated cell is shown (right). Scale bar, 120 μm. **d**, Evolution of the number of mutated cells in an inducible mini-colon after blue–light-mediated activation. Each dot represents a measurement every 15 min. The 2^nd^ order smoothing of the data is shown. **e**, Time-course quantitation of cell shedding (total protein content) into the lumen of OptoCre and control mini-colons after blue-light induced oncogenic recombination. *P = 0.0156; **P = 0.0033; ***P = 0.0003 (24 h), <0.0001 (96 h) (two-way ANOVA and Sidak’s multiple comparisons test; *n* = 3 mini-colons for each condition). Each point represents one mini-colon. Data represent mean ± SEM. **f**, Low- (left) and high-magnification (right) immunofluorescence images showing the presence of CD44 (magenta) and nuclei (blue) in a tumour-bearing mini-colon. White and grey arrowheads indicate early and advanced tumorigenic events, respectively. Scale bars, 120 μm (left) and 50 μm (right). Source Data

On the basis of previous evidence that small intestine cells can form stable tube-shaped epithelia through scaffold-guided organoid morphogenesis in microfluidic devices^9^, we next aimed to establish a ‘mini-colon’ constituted by OptoCre-AKP cells. By seeding colon cell suspensions in hydrogel-patterned microfluidic devices, we generated single-layered colonic epithelia spatially arranged into crypt- and lumen-like domains (Extended Data Fig. 2a). This spatial arrangement recapitulated the spatial distribution found in vivo, with stem and progenitor (SOX9^+^) cells located at the bottom of the crypt domains and more differentiated colonocytes (FABP1^+^) located in the upper crypt and lumen areas^17,18^ (Extended Data Fig. 2b). In contrast to conventional colon organoids, the lumen of these mini-colons was readily perfusable with fresh medium, enabling the removal of cell debris and extending their lifespan to several weeks without the need for passaging or tissue disruption (Extended Data Fig. 2a).

**Fig. 1:**
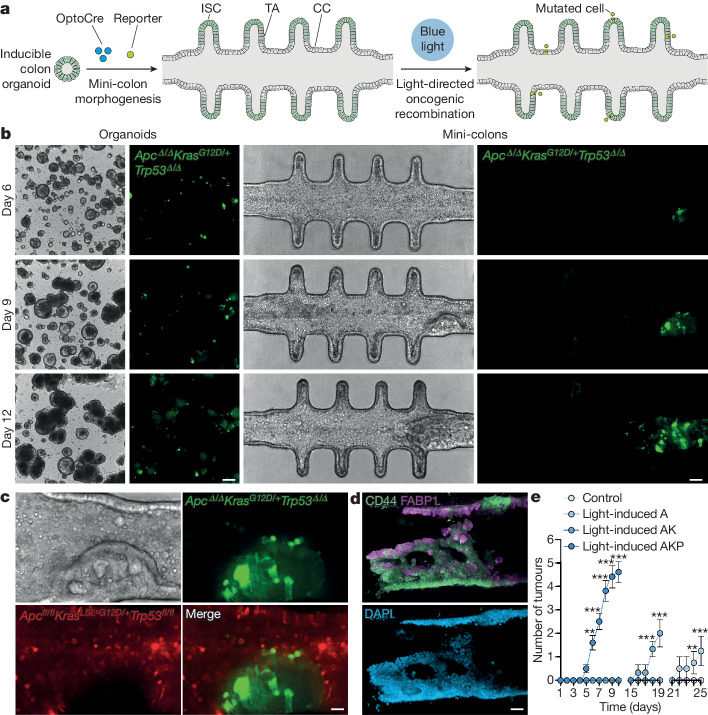
Spatiotemporally regulated de novo tumorigenesis in mini-colons. **a**, Schematic of the experimental workflow followed to induce tumorigenesis in mini-colons. CC, colonocyte; ISC, intestinal stem cell; TA, transit-amplifying cell. **b**, Bright-field and fluorescence images of time-course tumorigenesis experiments in conventional organoids and mini-colons. Fluorescence signal indicates oncogenic recombination. Scale bars, 200 μm (left) and 75 μm (right). **c**, Bright-field and fluorescence close-up images of a mini-colon tumour. The red and green signals correspond to healthy and mutated cells, respectively. Scale bar, 25 μm. **d**, Immunofluorescence images of a mini-colon tumour showing the presence of CD44 (top, green), FABP1 (top, magenta) and nuclei (bottom). Scale bar, 35 μm. **e**, Multiplicity of tumours emerged in mini-colons of the indicated genotypes after light-mediated oncogenic induction. Statistical analysis was performed using two-way analysis of variance (ANOVA) with Sidak’s multiple-comparison test; **P = 0.024 (day 6, AKP), **P = 0.0021 (day 24, A), ***P < 0.0001 (all other conditions). *n* = 5, 4, 3 and 10 mini-colons for the control, light-induced A, light-induced AK and light-induced AKP conditions, respectively. Data are mean ± s.e.m. Source Data

Once the healthy mini-colon system was established, we investigated its potential to capture tumour biology by inducing oncogenic recombination through blue-light illumination (Fig. 1a). To mimic the scenario found in vivo, we fine-tuned OptoCre activation to mutate only a small number of cells (<0.5% of the total population). Due to the stability and defined topology of the mini-colon, we easily detected the acquisition of AKP mutations at the single-cell level (GFP^+^ cells) and tracked their evolution over time (Extended Data Fig. 2c,d). This revealed that cell death is one of the earliest responses to oncogenic recombination, as mutated mini-colons displayed higher cell shedding rates compared with the controls (Extended Data Fig. 2e), with a large fraction of the mutated cells undergoing apoptosis (Supplementary Video 1). Nevertheless, some mutated cells escaped apoptosis and, after a quiescent period (24–72 h), started dividing at an accelerated pace (Extended Data Fig. 2d). In conventional organoid cultures, these fast-proliferating mutated cells did not lead to any overt tissular rearrangements (Fig. 1b), whereas, in the mini-colon system, they developed neoplastic structures over 5–10 days (Fig. 1b). Furthermore, these mini-colon neoplasias evolved from polyp-like to full-blown tumours, recapitulating in vivo tumorigenesis (Fig. 1b,c and Supplementary Videos 2 and 3).

**Extended Data Fig. 3:**
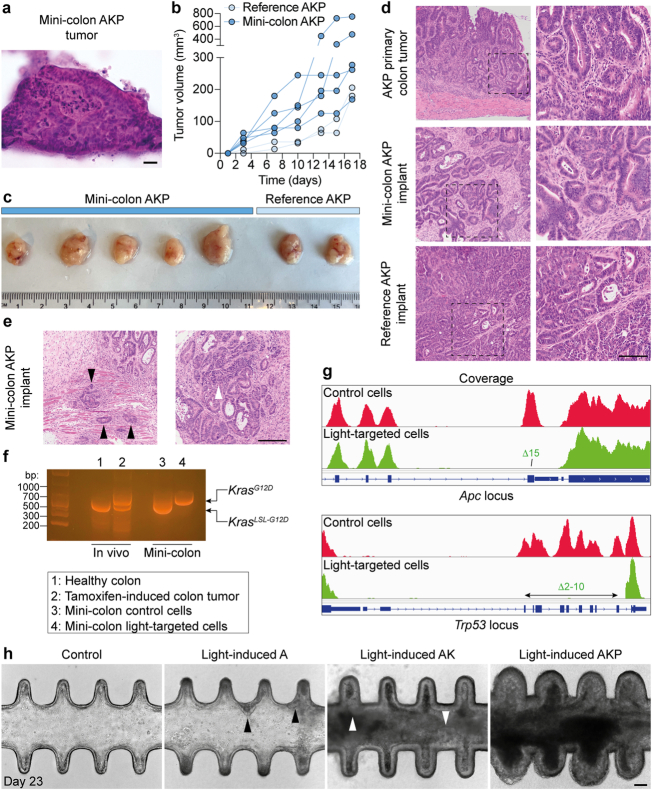
Oncogenic mutations induce full blown tumours in mini-colons. **a**, Hematoxylin and eosin staining of a mini-colon tumour section. Scale bar, 25 μm. **b**, Time-course growth of tumours produced by cells derived from mini-colon tumours upon subcutaneous transplantation in immunodeficient mice (*n* = 5 mice). As a reference, *bona fide* cancer cells from primary colon tumours are included. **c**, Image of the tumours at the endpoint of the experiment shown in panel b. **d**, Hematoxylin and eosin stainings of sections from the indicated tumour types. Zoomed-in areas (right) are indicated with a dashed square (left). Scale bar, 100 μm. **e**, Hematoxylin and eosin stainings of sections from mini-colon AKP implant tumours showing the presence of invading cancer cells (left, black arrowheads) and areas of cellular atypia (right, white arrowhead). Scale bar, 200 μm. **f**, Electrophoretic separation of PCR-amplified *KRAS*^*LSL*^ locus in the indicated samples. See Methods for more details on PCR design. **g**, Whole exome sequencing coverage in the indicated loci and cells. Missing exons in recombined cells are indicated. **h**, Brightfield images of mini-colons of the indicated genotypes 23 days after blue light exposure. Neoplastic and tumour structures are indicated with black and white arrowheads in A and AK mini-colons, respectively. By that time tumours have extended throughout the whole mini-colon tissue in the case of the AKP model, forming a dense mass of cancer cells. Scale bar, 75 μm. Source Data

Immunostaining analyses revealed that these tumours stemmed from CD44^high^ cells—a bona fide marker for cancer stem cells in vivo^19^—at the base of the epithelium (Fig. 1d, Extended Data Fig. 2f and Supplementary Video 4). Conversely, the bulk of the tumours was composed of cells with different degrees of differentiation, as revealed by the downregulation and upregulation of CD44 and FABP1, respectively (Fig. 1d and Supplementary Video 5). This indicated the existence of intratumour heterogeneity in the mini-colon, resembling the in vivo scenario^20^. Consistent with this, histopathological studies showed that these tumours displayed the histological organization characteristic of tubular adenomas (Extended Data Fig. 3a). To validate their cancerous nature, we performed transplantation experiments in immunodeficient mice and found that mini-colon-derived cancer cells formed tumours in vivo with undistinguishable efficiency from bona fide tumour-derived cancer cells (Extended Data Fig. 3b,c). Moreover, their histopathological structure was also comparable to the one displayed by primary tumours developed in the colon of AKP mice (Extended Data Fig. 3d) and included the presence of locally invasive nodules and areas with adenocarcinoma-like features (Extended Data Fig. 3e).

We confirmed through PCR and exome sequencing that tumour development in the mini-colon was directly associated with the expected mutations at the *Apc, Kras* and *Trp53* loci (Extended Data Fig. 3f,g). Consistent with this, using organoid lines with a reduced mutational burden (*Apc*^*fl/fl*^*Kras*^*LSL-G12D/+*^ (hereafter, AK) and *Apc*^*fl/fl*^ (hereafter, A)) produced longer latencies in tumour development in a dosage-dependent manner (Fig. 1e and Extended Data Fig. 3h), demonstrating that mini-colon tumorigenesis can be modulated by the number of oncogenic driver mutations. Collectively, these data show that the mini-colon system enables spatiotemporally controlled in vitro modelling of CRC tumorigenesis with a considerable degree of topobiological complexity.

## Context-dependent tumorigenic plasticity

**Fig. 2:**
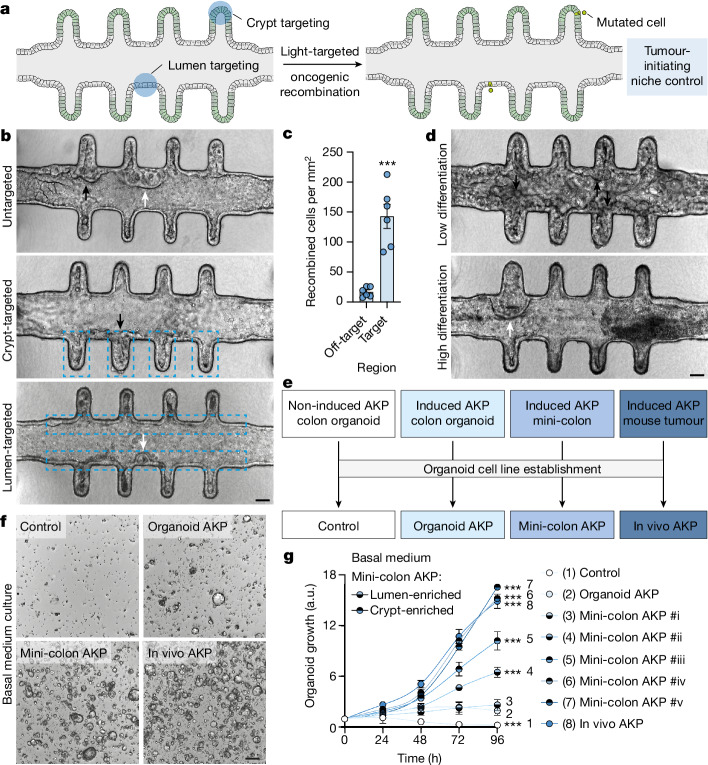
Mini-colons display context-dependent tumorigenic plasticity. **a**, Schematic of the experimental workflow followed to spatiotemporally target tumorigenesis in mini-colons. **b**, Bright-field images of mini-colons that have undergone untargeted (top), crypt-targeted (middle) and lumen-targeted (bottom) tumorigenesis. Targeted areas are indicated by dashed blue lines. The black and white arrows indicate tumours with compact and cystic morphologies, respectively. Scale bar, 75 μm. **c**, The oncogenic recombination efficiency in targeted and off-target areas in mini-colons. Statistical analysis was performed using two-tailed *t*-tests; ***P < 0.0001. *n* = 6 mini-colons per condition. Each point represents one mini-colon. **d**, Bright-field images of induced mini-colons cultured in low-differentiation (top, WENRNi) and high-differentiation (bottom, ENR) conditions. The black and white arrows indicate tumours with compact and cystic morphologies, respectively. Scale bar, 75 μm. **e**, Schematic of the different colon organoid lines generated in this work. **f**, Bright-field images of the indicated colon organoid lines cultured for 2 days in basal medium. Scale bar, 200 μm. **g**, Metabolic activity (measured using resazurin) of the indicated colon organoid lines cultured in basal medium for the indicated time. Numerical labelling (1–8) was used to facilitate cell line identification. Statistical analysis was performed using two-way ANOVA with Sidak’s multiple-comparison test; ***P = 0.0004 (control), ***P < 0.0001 (all other conditions). *n* = 3 cultures for each line. For **c** and **g**, data are mean ± s.e.m. Source Data

**Extended Data Fig. 4:**
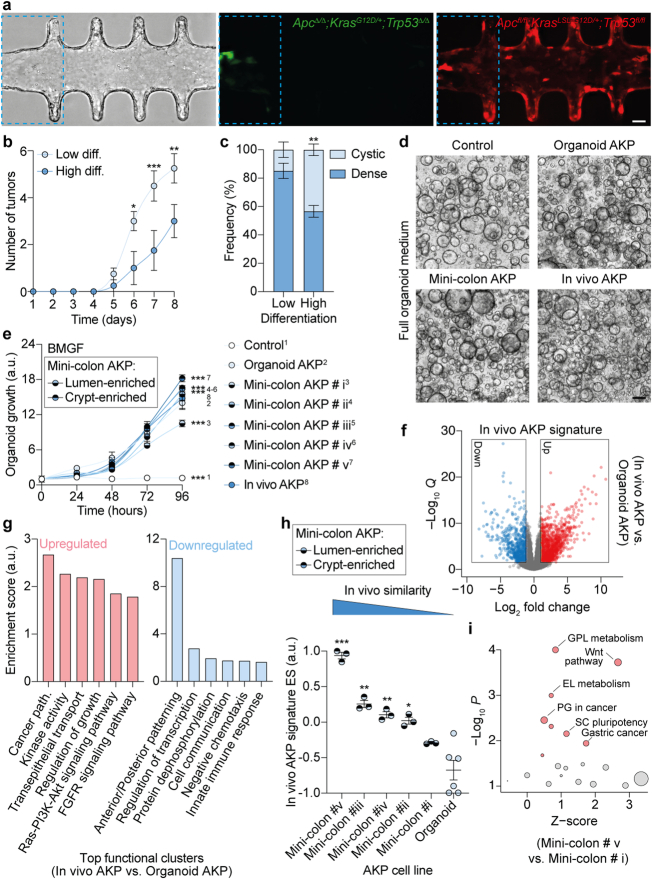
Mini-colon tumours display *in vivo*-like functional and transcriptional features. **a**, Brightfield and fluorescence images of a mini-colon where blue light exposure has been targeted to a specific area (dashed blue line). Red and green signals correspond to healthy and mutated cells, respectively. Images were taken 36 h after induction. Scale bar, 75 μm. **b**, Multiplicity of tumours emerged in mini-colons cultured in the indicated conditions. *P = 0.0122; **P = 0.0035; ***P = 0.0002 (two-way ANOVA and Sidak’s multiple comparisons test; *n* = 4 mini-colons for each condition). **c**, Distribution of tumour morphologies in mini-colons cultured in the indicated conditions. **P = 0.0024 (two-way ANOVA and Sidak’s multiple comparisons test; *n* = 4 mini-colons for each condition). **d**, Brightfield images of the indicated colon organoid lines cultured for 3 days in full organoid medium. Scale bar, 200 μm. **e**, Metabolic activity (measured using resazurin) of the indicated colon organoid lines cultured in BMGF medium for the indicated time. Numeric labelling (1-8) is used to facilitate cell line identification. ***P = 0.0002 (mini-colon AKP #iii), <0.0001 (all other conditions) (two-way ANOVA and Sidak’s multiple comparisons test; *n* = 3 cultures for each line). **f**, Volcano plot of the differentially expressed genes between “in vivo” and “organoid AKP” cell lines. **g**, Top enriched functional clusters in the differentially expressed genes identified in panel f. **h**, Enrichment of the “in vivo AKP” transcriptional signature identified in panel f across the different “mini-colon” and “organoid AKP” lines. *P = 0.0137; **P = 0.0032 (#iii), 0.0075 (#iv); ***P = 0.0001 (Brown-Forsythe ANOVA and Dunnett’s T3 multiple comparisons test (two-sided), *n* = 6 and 3 cultures for “organoid AKP” and the rest of cell lines, respectively). Each dot represents one culture. **i**, Main enriched functional terms in the differentially expressed genes between “mini-colon AKP” lines # i and # v. Significant terms are highlighted in red (one-sided Fisher’s exact test, adjusted *P* values). In **b**, **c**, **e**, and **h**, data represent mean ± SEM. GPL, glycerophospholipid; EL, ether lipid; PG, proteoglycans; SC, stem cell. Source Data

Careful examination of induced mini-colons revealed consistent morphological differences among tumours according to their initiation site, with prominent dense or cystic internal structures arising from the crypt and the luminal epithelium, respectively (see below; Fig. 2b (top)). As mini-colons comprise different types of cells along the crypt–lumen axis (Extended Data Fig. 2b), we leveraged the spatial resolution provided by OptoCre to investigate whether the initiating cell niche conditioned the morphological and functional features of nascent tumours. To spatially control AKP mutagenesis, we coupled the mini-colon to a photomask restricting blue-light exposure to specific regions of the colonic epithelium (Fig. 2a), which provided low off-target recombination rates (around 8.5%) (Fig. 2b,c and Extended Data Fig. 4a). Here again, dense and cystic tumours developed when crypt and lumen epithelia, respectively, were mutationally targeted by blue light (Fig. 2b). To confirm that this was associated with the differentiation status of the tumour-initiating cell, we cultured mini-colons in either low- or high-differentiation medium before oncogenic induction to shift the proportions of (un)differentiated cells. Low-differentiation conditions produced mini-colons with thicker epithelia, early tumour development and a reduced fraction of cystic tumours (Fig. 2d and Extended Data Fig. 4b,c). Conversely, high-differentiation conditions produced mini-colons with thinner epithelia, delayed tumour formation and increased cystic tumour frequency (Fig. 2d and Extended Data Fig. 4b,c). These results indicate that the different environments of the mini-colon can shape tumour fate.

To evaluate the functional repercussions of the tumour-initiating niche, we isolated cancer cells from mini-colons enriched in either crypt- or lumen-derived tumours and established organoid cell lines (termed mini-colon AKP) (Fig. 2e). As a control, we generated AKP mutant organoids by shining blue light onto inducible organoids and kept these mutants in parallel with their mini-colon equivalents, doing the required passages on confluency (termed organoid AKP) (Fig. 2e). We also established organoid cultures from AKP colon tumours extracted from tamoxifen-treated *Cdx2-cre*^*ERT2*^ AKP mice (termed in vivo AKP) (Fig. 2e). Notably, in contrast to mini-colons, none of these three types of mutant AKP lines were morphologically distinguishable from healthy non-mutated cells when cultured as organoids (Fig. 1b and Extended Data Fig. 4d). When we cultured these organoids in basal medium depleted of growth factors (BM; Methods), both in vivo and crypt tumour-derived mini-colon AKP organoids preserved their proliferative potential (Fig. 2f,g). Conversely, organoid and lumen tumour-enriched mini-colon AKP lines displayed significantly reduced proliferation rates (Fig. 2f,g). This was not due to intrinsic cycling defects in any of the organoid lines tested, as these differences were not observed in standard cancer organoid medium (BMGF; Methods and Extended Data Fig. 4e). As expected, healthy organoids did not grow in any of these conditions (Fig. 2f,g and Extended Data Fig. 4e). Collectively, these results show that there are context-dependent factors aside from the founding AKP mutations that condition the growth potential of AKP cells. They also indicate that the cells derived from mini-colon crypt tumours recapitulate the growth properties of in vivo CRC cells more faithfully than conventional organoids.

**Extended Data Fig. 5:**
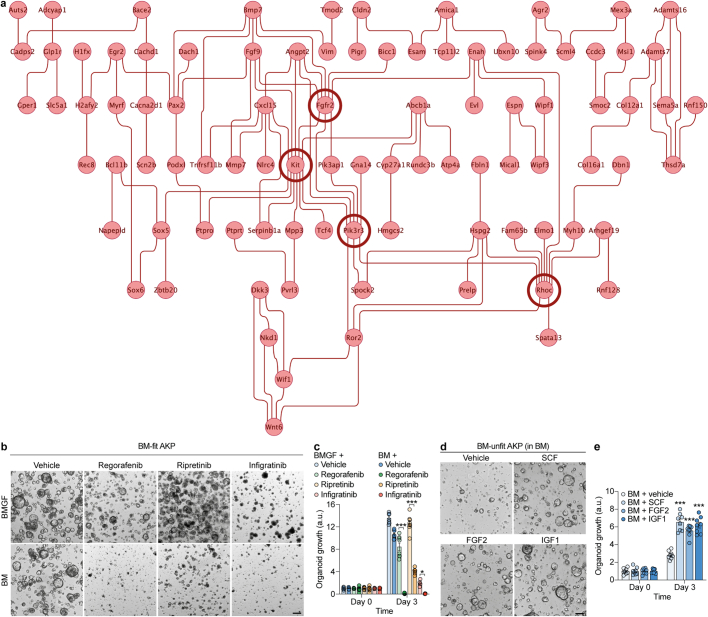
Tumorigenesis in the mini-colon leads to enhanced RTK signalling promoting growth factor independence. **a**, Gene interaction network of the overlapping genes that are upregulated in AKP lines with high-proliferation potential in BM (“in vivo AKP”, “mini-colon AKP” #v) when compared to low-growth counterparts (“organoid AKP”, “mini-colon AKP” #i). Network hubs are highlighted with circles. **b**, Brightfield images of “mini-colon AKP” #v organoids cultured for 3 days with the indicated media and compounds. Scale bar, 200 μm. **c**, Metabolic activity (measured using resazurin) of “mini-colon AKP” #v organoids cultured in the indicated conditions. *P = 0.0236; ***P < 0.0001 (two-way ANOVA and Sidak’s multiple comparisons test; *n* = 4 cultures for infigranib and 8 for the rest of conditions). **d**, Brightfield images of “organoid AKP” cells cultured for 3 days in BM with the indicated growth factors. Scale bar, 200 μm. **e**, Metabolic activity (measured using resazurin) of “organoid AKP” cells cultured in the indicated conditions. ***P < 0.0001 (two-way ANOVA and Sidak’s multiple comparisons test; *n* = 8 cultures for each condition). In **c** and **e**, each point represents one well of organoids and data represent mean ± SEM. Source Data

To investigate the molecular programs underpinning these observations, we profiled the transcriptome of the different AKP lines using RNA sequencing (RNA-seq). We first characterized the differences between the two AKP lines derived from conventional systems, in vivo and organoid AKP cells, which also had the biggest disparity in growth potential (Fig. 2g). According to our previous experiments, in vivo AKP cells upregulated many genes involved in canonical cancer pathways and the promotion of cell growth (Extended Data Fig. 4f,g). Conversely, these cells downregulated genes associated with cell differentiation, patterning and transcriptional regulation (Extended Data Fig. 4f,g). To evaluate whether mini-colon AKP cells recapitulated this in vivo AKP transcriptional signature, we performed single-sample gene set enrichment analysis (GSEA) across all of the cell lines. Here, most of the mini-colon AKP lines outscored their organoid AKP counterparts, especially those derived from crypt tumours (Extended Data Fig. 4h). To investigate the transcriptional divergence between crypt- and lumen-enriched mini-colon AKP cells, we compared the lines with the highest (#v, crypt-enriched) and lowest (#i, lumen-enriched) in vivo AKP signature score (Extended Data Fig. 4h). These analyses revealed that crypt-derived mini-colon AKP cells upregulated genes involved in WNT signalling, stem cell pluripotency, lipid metabolism and other pathways involved in cancer (Extended Data Fig. 4i). To identify the potential drivers of growth factor independence among these, we searched for overlaps between AKP lines with high growth potential in BM (in vivo AKP, mini-colon AKP #v). We found that the latter overexpressed a collection of genes that is involved in the activation of MAPK cascades, including receptor tyrosine kinases (RTKs), G-protein-coupled receptors and soluble factors (Extended Data Fig. 5a). We therefore theorized that these cells were engaging a surplus of MAPK signalling that gave them a greater fitness under growth-factor-poor conditions. To validate this idea, we tested their response to a panel of inhibitors, which confirmed that the growth of AKP lines in BM heavily relied on signals from RTKs (Extended Data Fig. 5b,c; regorafenib), including KIT (Extended Data Fig. 5b,c; ripretinib) and FGF receptors (Extended Data Fig. 5b,c; infigratinib). Corroborating this, the ligands for these RTKs (SCF, FGF2) and others involved in colonocyte clonogenicity (IGF1)^21^ could enhance the growth of the AKP lines with poor proliferation potential in BM (Extended Data Fig. 5d,e). Importantly, all of these dependencies were either reduced or not detectable in conventional CRC organoid medium (BMGF) (Extended Data Figs. 4e and 5b,c). Taken together, these data indicate that the mini-colon is a plastic system in which context-dependent factors can drive different functional features in CRC cells, including the engagement of ancillary RTK signals that boost their growth potential in challenging environments.

## Intra- and intertumour heterogeneity

**Fig. 3:**
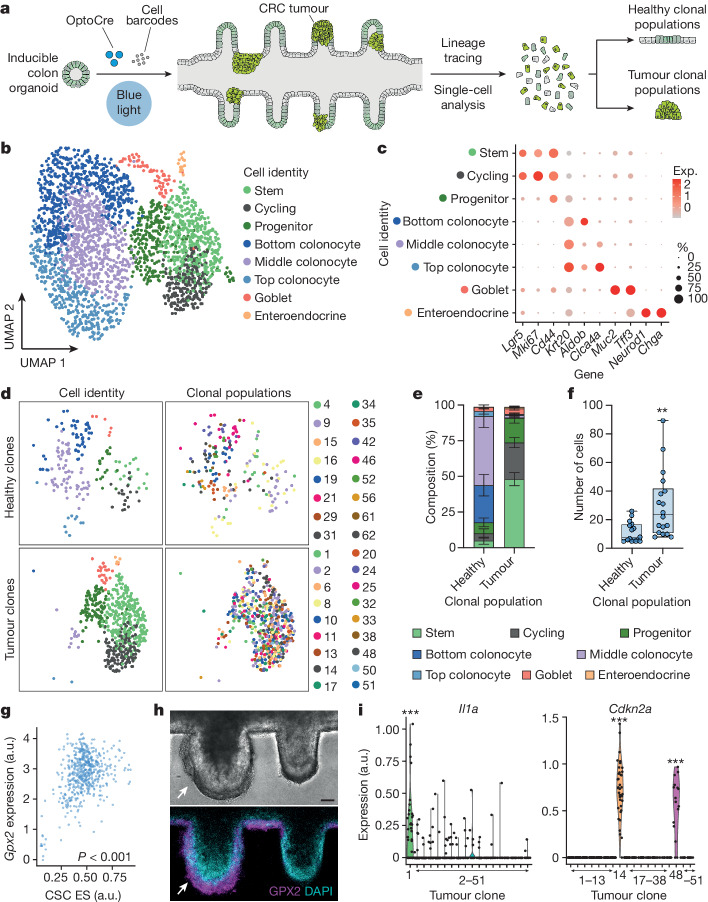
Mini-colons support intratumour and intertumour heterogeneity. **a**, Schematic of the experimental workflow followed for single-cell and lineage-tracing analysis of mini-colons. **b**, Unsupervised uniform manifold approximation and projection (UMAP) clustering of the main cell types in mini-colons 7 days after tumorigenic induction. **c**, The expression (Exp.) of representative cell-type-specific markers in the different cell populations comprising mini-colons. **d**, Unsupervised clustering (UMAP) of healthy (top) and tumour (bottom) clonal populations in mini-colons. The cell type (left; colour coded as in **b**) and clonal identity (right) are indicated. **e**, The relative cell type abundance in healthy and tumour mini-colon clonal populations. Data are mean ± s.e.m. *n* = 16 and 18 for healthy and tumour clones, respectively. **f**, Healthy and tumour mini-colon clonal population sizes. Statistical analysis was performed using two-tailed Mann–Whitney *U*-tests; **P = 0.0011. *n* = 16 and 18 for healthy and tumour clones, respectively. The box plots show the median (centre lines), the first and third quartiles (box limits) and the minimum and maximum values (whiskers). Each point represents one clonal population. **g**, The correlation between *Gpx2* expression and cancer stem cell transcriptional signature enrichment (*Cd44*, *Lgr5*, *Sox9*). Statistical analysis was performed using two-sided Pearson correlation tests; *P* < 0.0001. *n* = 540 cells. Each point represents one cell. CSC, cancer stem cell; ES, enrichment score. **h**, Bright-field and immunofluorescence images showing the abundance of GPX2 (magenta) and nuclei (cyan) in healthy (right) and tumour (left, indicated by arrows) crypts in a mini-colon. Scale bar, 35 μm. **i**, Expression of the indicated genes in the indicated tumour clones. Statistical analysis was performed using two-sided Wilcoxon rank-sum tests; ***P = 1.77 × 10^−17^ (*Il1a*, clone 1), 1.00 × 10^−78^ (*Cdkn2a*, clone 14), 3.67 × 10^−22^ (*Cdkn2a*, clone 48). *n* = 540 cells. Each point represents one cell. Source Data

**Extended Data Fig. 6:**
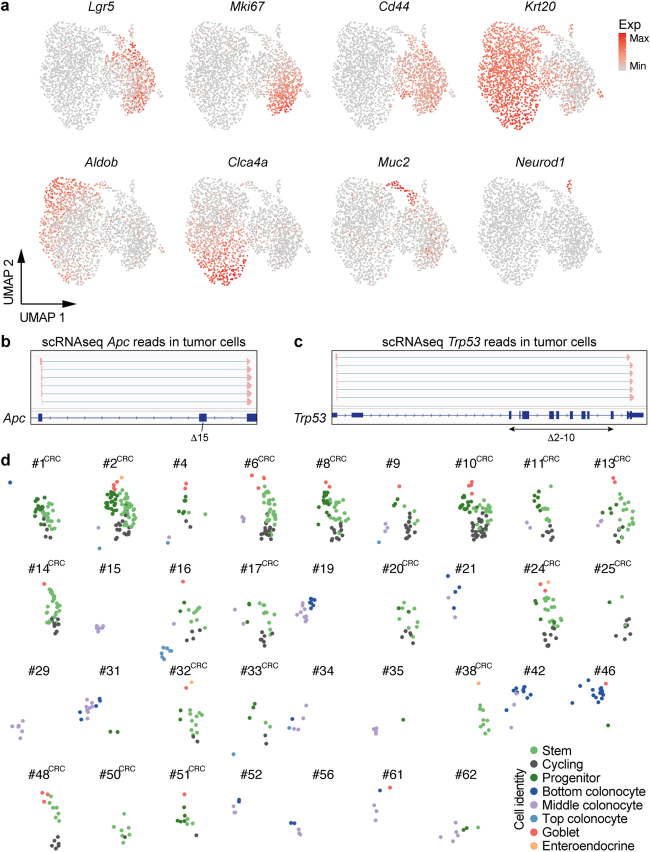
Mini-colons comprise a complex cellular ecosystem. **a**, Expression distribution of cell–type-specific markers across mini-colon cells. Cell-type labels can be found in Fig. 3b. **b**, Examples of single-cell RNA reads capturing exon-exon junctions that reveal the expected oncogenic recombination in *Apc*. **c**, Examples of single-cell RNA reads capturing exon-exon junctions that reveal the expected oncogenic recombination in *Trp53*. **d**, Unsupervised UMAP clustering of the main cell types found in each of the healthy and tumour clonal populations found within the mini-colon. Tumour clones carry the “CRC” label. UMAP structure corresponds to the one shown in Fig. 3b.

We hypothesized that the diversity observed in tumour morphology and growth potential reflected clonally distinct tumour types being initiated in the mini-colon. To validate this idea, we performed single-cell transcriptomic profiling of tumour-bearing mini-colons incorporating a genetic cell barcoding system^22^ to preserve clonal information (Fig. 3a). On the basis of bona fide transcriptional markers, mini-colons comprised eight major cell types that were segregated into undifferentiated, absorptive and secretory lineages (Fig. 3b). Undifferentiated (*Krt20*^−^) cells included stem (*Lgr*5^+^), actively proliferating (*Mki67*^+^) and progenitor (*Sox9*^+^*Cd44*^+^) cells (Fig. 3b,c and Extended Data Fig. 6a). Mature (*Krt20*^+^) absorptive colonocytes constituted the largest fraction of the mini-colon, and included bottom, middle and top colonocytes based on zonation markers^23^ (such as *Aldob*, *Iqgap2* and *Clca4a*) (Fig. 3b,c and Extended Data Fig. 6a). Mucus-producing goblet cells (*Muc2*^+^) and hormone-releasing enteroendocrine cells (*Neurod1*^+^) constituted the secretory compartment (Fig. 3b,c and Extended Data Fig. 6a). Collectively, this diverse in vivo-like cell composition indicates that mini-colons provide a physiologically relevant context for conducting oncogenesis studies.

To determine the clonal identities across the mini-colon, we compared the genetic barcodes among cells and detected 83 clonal populations. We then discarded small (<5 cells) clones and identified cells containing reads corresponding to the mutated versions of *Apc* and *Trp53* (Extended Data Fig. 6b,c). These bona fide tumour cells distinguished tumour clonal populations (18 classified) from healthy counterparts (16 classified) (Methods and Extended Data Fig. 6d). On average, healthy clonal populations consisted of around 18% undifferentiated cells, which gave rise to the remaining approximately 82% of absorptive colonocytes and secretory cells (Fig. 3d,e). Conversely, mini-colon tumours were mostly formed by undifferentiated cells (~92%), with sparsely present colonocytes and secretory cells (Fig. 3d,e). Tumour cells also formed larger clonal populations compared with their healthy counterparts (Fig. 3f). These cell proportions are well aligned with the ones commonly observed in vivo^24,25^.

**Extended Data Fig. 7:**
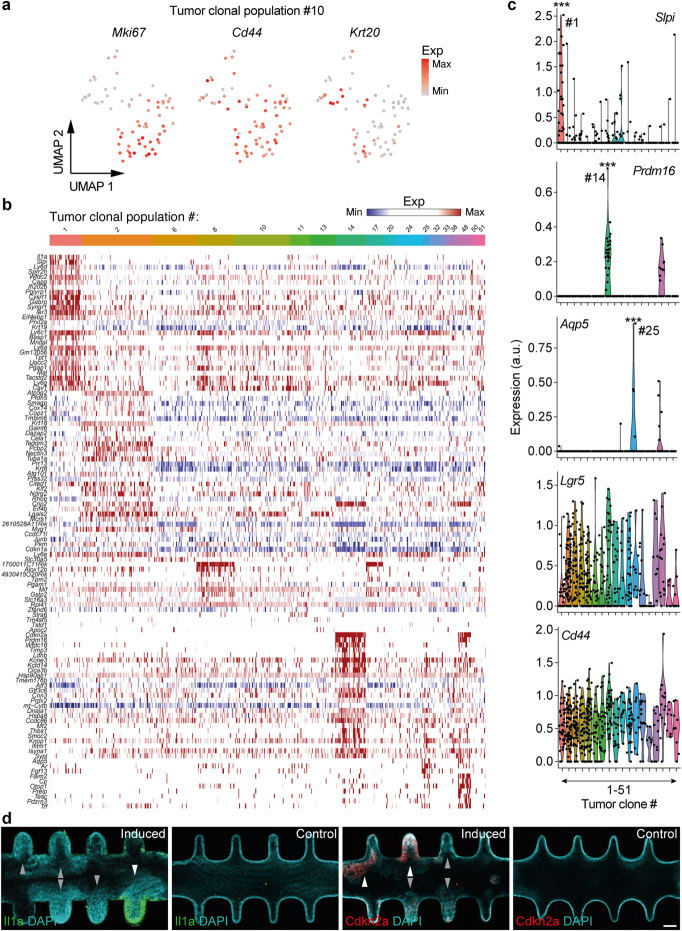
Mini-colons display intra- and inter-tumour diversity. **a**, Expression distribution of proliferation (*Mki67*), stemness (*Cd44*), and differentiation (*Krt20*) markers within a single clonal tumour population. **b**, Heatmap of the genes showing the strongest (*P* < 10^−5^) differential expression across mini-colon tumours. The tumour clonal population is indicated on top. **c**, Expression of the indicated genes in the indicated tumour clones. ***P = 2.74·10^−14^ (*Slpi*, clone #1), 4.06·10^−50^ (*Prdm16*, clone #14), 1.05·10^−13^ (*Aqp5*, clone #25) (two-sided Wilcoxon rank-sum test; *n* = 540 cells). Each point represents one cell. **d**, Immunofluorescence images showing the expression of *Il1a* (left, green), *Cdkn2a* (right, red), and the presence of nuclei (cyan) in tumour-bearing and control mini-colons. White and grey arrowheads indicate positive and negative tumours, respectively, in terms of marker expression. Scale bar, 100 μm.

Analyses of the internal structure of single clonal tumours showed that they comprised a non-homogeneous collection of cells with diverse proliferation, stemness and differentiation markers (Extended Data Fig. 7a). Such intratumour heterogeneity reflects the complexity of mini-colon tumours, consistent with our immunostaining data (Fig. 1d). To investigate the mechanisms orchestrating cancer stemness and tumour development, we analysed the transcriptional differences between differentiated (*Krt20*^+^*Apoc2*^+^*Fabp2*^+^) and stem (*Lgr5*^+^*Cd44*^+^*Sox9*^+^) cancer cells within tumours. We found that *Gpx2*, a glutathione peroxidase recently linked to CRC malignant transformation^24^, strongly correlated with the stemness potential of mini-colon cancer cells (Fig. 3g). Consistent with this, we observed that GPX2 protein was particularly enriched in the basal cells of mini-colon tumours (Fig. 3h).

**Extended Data Fig. 8:**
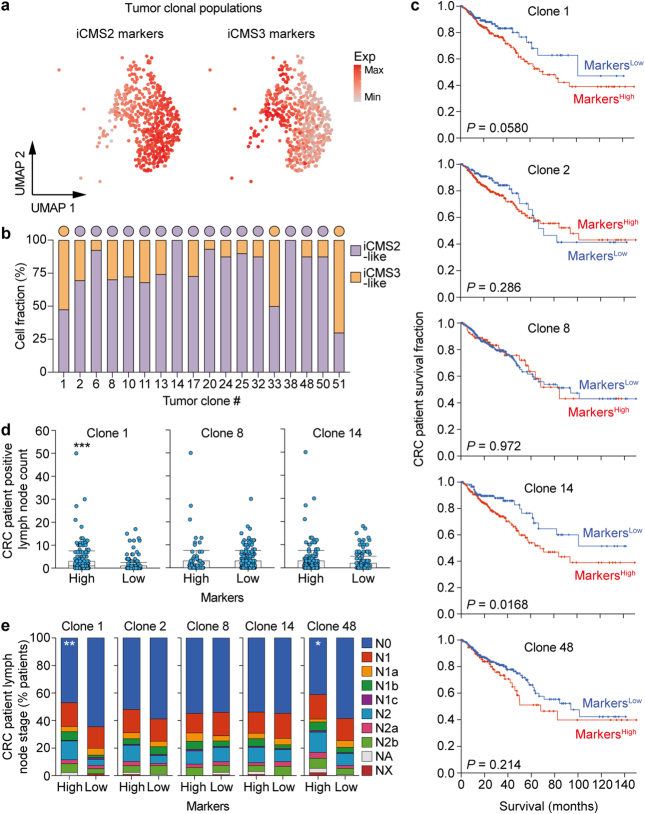
The tumour heterogeneity in the mini-colon is relatable to the human context. **a**, Expression distribution of intrinsic consensus molecular subtype (iCMS) signatures across tumour cells in the mini-colon. Cell-type labels can be found in Fig. 3d. Exp, expression. **b**, Fraction of cells within each tumour clone in the mini-colon classified in each iCMS group. **c**, Survival of CRC patients from the TCGA database according to the expression of the indicated tumour clone-specific markers. The logrank test *P* value is indicated (*n* = 375 patients). **d**, Presence of cancer cells in lymph nodes from CRC patients from the TCGA database according to the expression of the indicated tumour clone-specific markers. ***P = 2.714·10^−5^ (two-sided Wilcoxon test; *n* = 375 patients). **e**, Lymph node staging in CRC patients from the TCGA database according to the expression of the indicated tumour clone-specific markers. *P = 0.0222; **P = 4.923·10^−3^ (two-sided Chi-squared test; *n* = 375 patients). Source Data

To examine whether mini-colons could produce different types of tumours, we next compared the transcriptional profiles of the different tumour clones. Even though all tumour-initiating cells carried the same founding AKP mutations and shared many molecular features, we found clear diversity across mini-colon tumours (Extended Data Fig. 7b). For example, the expression of the interleukin *Il1a* and leukocyte peptidase inhibitor *Slpi* revealed the presence of tumours with an inflammatory-like profile (Fig. 3i and Extended Data Fig. 7b,c). *Cdkn2a* (encoding tumour suppressors p14 and p16) and *Prdm16* were exclusively expressed by tumours seemingly insensitive to these cell cycle arrest genes given their *Ki67*^*+*^ nature (Fig. 3i and Extended Data Figs. 6a and 7b,c). *Aqp5*, an aquaporin inductor of gastric and colon carcinogenesis^26^, marked specific tumours able to produce the oncogenesis-promoting fibroblast growth factor FGF13 (Extended Data Fig. 7b,c). Together with other markers (Extended Data Fig. 7b) and corroborations at the protein level (Extended Data Fig. 7d), these data indicate that a variety of tumour subtypes can be generated in the mini-colon, arguably due to tumour-niche-intrinsic and/or environmental factors. This probably accounts for the observed differences among mini-colon AKP cell lines (Fig. 2g and Extended Data Fig. 4h). Importantly, we found that this diversity was relatable to the human context. For example, mini-colons generated tumours with transcriptional profiles representing both iCMS2- and iCMS3-like subtypes^27^ (Extended Data Fig. 8a,b) that were associated with a wide range of aggressiveness profiles (Extended Data Fig. 8c) and correlated with different extents of lymph node colonization (Extended Data Fig. 8d,e) when cross-compared with transcriptomic data from the TCGA collection of patients with CRC. Collectively, these findings demonstrate that the mini-colon is a complex cellular ecosystem that recreates both healthy and cancer cell diversity.

## Screening of tumorigenic factors

**Fig. 4:**
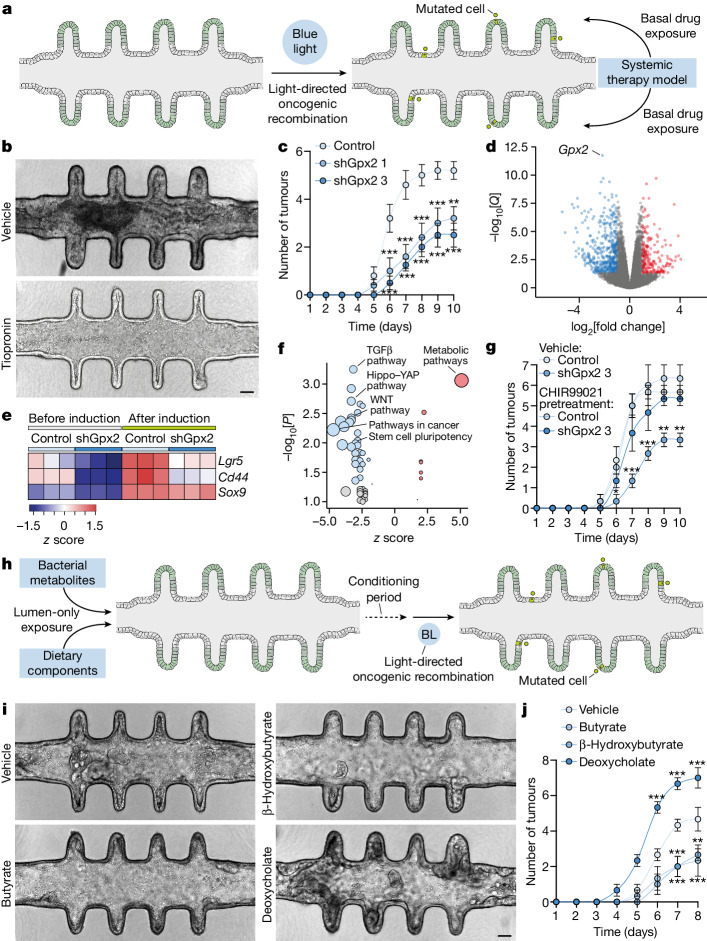
Mini-colons allow physiologically relevant screening of tumorigenic factors. **a**, The experimental workflow for systemic therapy modelling. **b**, Bright-field images of mini-colons treated with vehicle or tiopronin after tumorigenic recombination. Images correspond to 6 days after induction. Scale bar, 75 μm. **c**, The multiplicity of tumours emerged in mini-colons of the indicated genotype after oncogenic induction. Statistical analysis was performed using two-way ANOVA with Sidak’s multiple-comparison test; **P = 0.0034, ***P = 0.0007 (days 6 and 9, sh*Gpx2* 1), ***P < 0.0001 (all other conditions). *n* = 5, 5 and 4 mini-colons for control, shGpx2 1 and shGpx2 3, respectively. **d**, Differentially expressed genes after *Gpx2* knockdown in light-induced AKP tumour cells. **e**, Expression of the indicated genes in colonocytes of the indicated genotypes before and after oncogenic recombination. The colour scale shows the *z* score. **f**, The main enriched functional terms after *Gpx2* knockdown in light-induced AKP tumour cells. Significant terms are highlighted in blue or red, as determined using one-sided Fisher’s exact tests, with gene expression adjusted *P*-values (Benjamini-Hochberg correction). **g**, The multiplicity of tumours emerged in mini-colons of the indicated genotype under the indicated pretreatment (2 days before oncogenic induction). Statistical analysis was performed using two-way ANOVA with Sidak’s multiple-comparison test; *P = 0.0274, **P = 0.0033 (days 9 and 10), ***P < 0.0001 (days 7 and 8). *n* = 3 mini-colons for each condition. **h**, The experimental workflow for microbiota and dietary pattern modelling. BL, blue light. **i**, Bright-field images of mini-colons treated with the indicated metabolites. Images correspond to 7 days after tumorigenic induction. Scale bar, 75 μm. **j**, The multiplicity of tumours emerged in mini-colons treated with the indicated metabolites. Statistical analysis was performed using two-way ANOVA with Sidak’s multiple-comparison test; **P = 0.0080, ***P = 0.0008 (days 7 and 8), ***P < 0.0001 (day 6). *n* = 3 mini-colons for each condition. For **c**, **g** and **j**, data are mean ± s.e.m. Source Data

**Extended Data Fig. 9:**
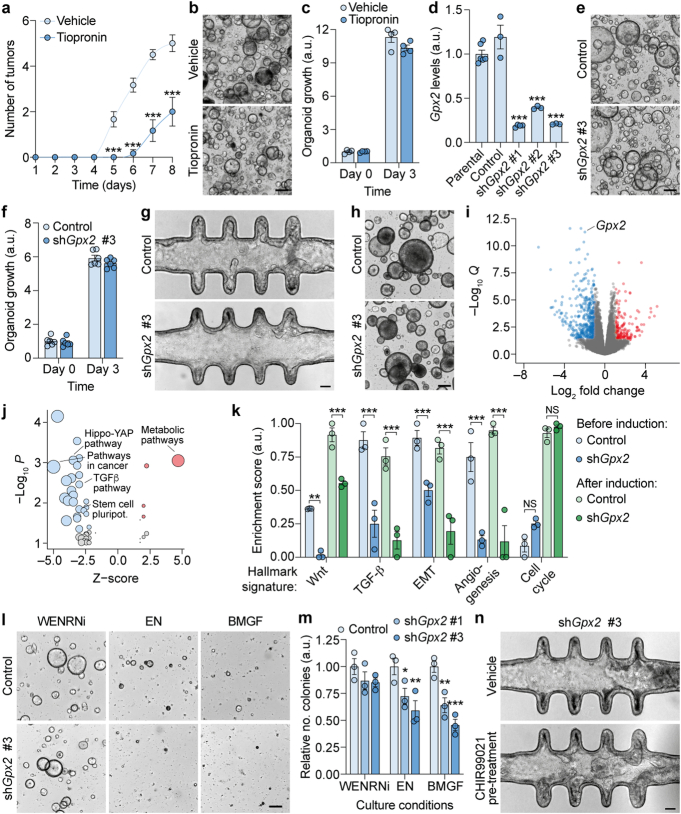
Gpx2 regulates colonocyte stemness and tumorigenesis. **a**, Multiplicity of tumours emerged in mini-colons treated with the indicated compound upon oncogenic induction. ***P = 0.0001 (day 5), <0.0001 (all other conditions) (two-way ANOVA and Sidak’s multiple comparisons test; *n* = 6 mini-colons for each condition). **b**, Brightfield images of colon organoids treated with the indicated compound after tumorigenic recombination. Images correspond to 3 days after induction. Scale bar, 200 μm. **c**, Metabolic activity (measured using resazurin) of organoids cultured in the indicated conditions and times after oncogenic recombination. No significant differences (two-way ANOVA and Sidak’s multiple comparisons test; *n* = 4 cultures for each condition). **d**, qRT-PCR based quantitation of *Gpx2* mRNA in the indicated cell lines. ***P < 0.0001 (one-way ANOVA and Tukey’s multiple comparisons test; *n* = 6, 4, and 3 organoid cultures for parental, sh*Gpx2* #1, and the rest of the lines, respectively). **e**, Brightfield images of non-induced colon organoids of the indicated genotypes after 3 days of culture. Scale bar, 200 μm. **f**, Metabolic activity (measured using resazurin) of non-induced colon organoids of the indicated genotypes at the indicated times. No significant differences (two-way ANOVA and Sidak’s multiple comparisons test; *n* = 6 cultures for each condition). **g**, Brightfield images of mini-colons of the indicated genotypes after tumorigenic recombination. Images correspond to 6 days after induction. Scale bar, 75 μm. **h**, Brightfield images of colon organoids of the indicated genotypes after tumorigenic recombination. Images correspond to 6 days after induction. Scale bar, 200 μm. **i**, Volcano plot showing the differentially expressed genes upon *Gpx2* knockdown in non-transformed colon cells. **j**, Bubble plot showing the main enriched functional terms in the differentially expressed genes upon *Gpx2* knockdown in non-transformed colon cells. Significant terms are highlighted in either blue (downmodulated) or red (upmodulated) (one-sided Fisher’s exact test, adjusted *P* values). **k**, Enrichment of the indicated hallmark signatures from the MSigDB in the indicated cell lines. **P = 0.0013; ***P = 0.0008 (Wnt), 0.0003 (EMT, before recombination), <0.0001 (all other conditions); NS, not significant (one-way ANOVA and Tukey’s multiple comparisons test; *n* = 3 cultures for each condition). **l**, Colony assay images of non-induced colon organoids of the indicated genotypes after 3 days of culture in the indicated media conditions. Scale bar, 200 μm. **m**, Clonogenic capacity of non-induced colon organoids of the indicated genotypes after 3 days of culture in the indicated media conditions. *P = 0.0219; **P = 0.0012 (EN, sh*Gpx2* #3), 0.0036 (BMGF, sh*Gpx2* #1); ***P < 0.0001 (two-way ANOVA and Dunnet’s multiple comparisons test; *n* = 3 cultures for each condition). **n**, Brightfield images of *Gpx2* knockdown mini-colons that had undergone the indicated pre-treatment before tumorigenic recombination. Images correspond to 7 days after tumour induction. Scale bar, 75 μm. In **a**, **c**, **d**, **f**, **k**, and **m**, each point represents one well of organoids and data represent mean ± SEM. Source Data

The longevity, experimental flexibility and tumour formation dynamics of mini-colons provides an unparalleled in vitro set-up for conducting tumorigenesis assays. We therefore next used mini-colons as screening tools for identifying molecules with a prominent role in tumour development. As our single-cell RNA-seq (scRNA-seq) analyses revealed *Gpx2* overexpression in cancer stem cells (Fig. 3g,h), we probed its functional relevance by adding the glutathione peroxidase inhibitor tiopronin^28^ to the basal medium reservoirs of mini-colons right after blue-light-induced AKP mutagenesis (Fig. 4a). Basal application of the drug provides ubiquitous exposure on the mini-colon basolateral domain, mimicking a systemic therapy model (Fig. 4a). By the time control mini-colons developed full-blown tumours, tiopronin-treated counterparts were largely tumour-free with a healthy colonic epithelium (Fig. 4b and Extended Data Fig. 9a). This was not due to the mere reduction in proliferative activity, as tiopronin had a minor impact on organoid growth (Extended Data Fig. 9b,c). As tiopronin targets several glutathione peroxidases, we corroborated the specific implication of GPX2 in tumour initiation by knocking down its transcript (Extended Data Fig. 9d). These knockdown cells showed no detectable defects in terms of organoid morphology or proliferation in unchallenged conditions (Extended Data Fig. 9e,f). However, after blue-light-mediated oncogenic recombination, GPX2-deficient mini-colons developed tumours with reduced kinetics and multiplicity (Fig. 4c and Extended Data Fig. 9g), recapitulating the results obtained with tiopronin (Fig. 4b and Extended Data Fig. 9a). Importantly, mini-colons were instrumental for these findings, as conventional organoid cultures cannot reveal differences in tumour-forming abilities (Extended Data Fig. 9b,h).

To gain molecular insights into the mechanism engaged by GPX2, we performed RNA-seq analysis of *Gpx2*-knockdown cells both before and after oncogenic recombination. These analyses revealed that GPX2 deficiency remodels the colonocyte transcriptome in both healthy (Extended Data Fig. 9i) and tumorigenic (Fig. 4d) conditions (Supplementary Tables 1 and 2). This included the downmodulation of canonical markers associated with both healthy and cancer cell stemness, such as *Lgr5* and *Cd44* (Fig. 4e). By contrast, markers of proliferative progenitor cells, such as *Sox9*, remained unchanged (Fig. 4e). Consistent with this, *Gpx2* abrogation led to the repression of transcriptional programs implicated in stem cell pluripotency, including the WNT, Hippo–YAP and TGFβ pathways, as well as epithelial–mesenchymal transition and other processes involved in cancer cell fitness (Fig. 4f and Extended Data Fig. 9j,k). Conversely, transcriptional programs associated with proliferation were not affected, consistent with our observations in cell culture (Extended Data Fig. 9e,f,k). These findings indicate that the inhibition of GPX2 downmodulates colonocyte stemness, which probably accounts for the reduced tumorigenic potential observed in the mini-colon after oncogenic recombination. Supporting this, we found that non-transformed GPX2-deficient cells displayed reduced clonogenic capacity in medium deprived of exogenous WNT signals (Extended Data Fig. 9l,m). Furthermore, the enhancement of WNT signalling through pretreatment of mini-colons with CHIR99021 for 2 days before oncogenic induction rescued the tumorigenic potential of *Gpx2-*knockdown cells (Fig. 4g and Extended Data Fig. 9n). Collectively, these data uncover GPX2 as a key regulator of colon stemness and tumorigenesis, shedding light on lingering questions spurred by the recent discovery of its association with the malignant progression of human CRC^24^.

**Extended Data Fig. 10:**
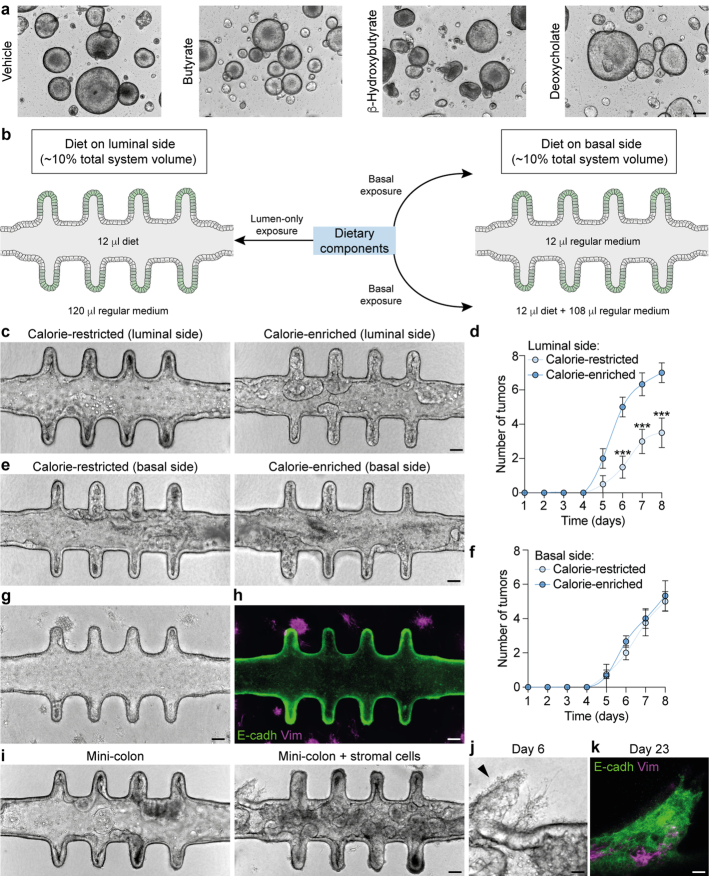
Mini-colons provide experimental versatility and resolution to tumorigenic studies. **a**, Brightfield images of colon organoids treated with the indicated bacterial metabolites. Images correspond to 5 days after oncogenic induction. Scale bar, 200 μm. **b**, Schematic of the experimental setup used to evaluate the relevance of luminal access in tumorigenic studies. **c**, Brightfield images of mini-colons treated with the indicated diets according to experimental setup displayed in panel b (left). Images correspond to 6 days after tumorigenic induction. Scale bar, 75 μm. **d**, Multiplicity of tumours emerged in mini-colons treated with the indicated diets according to experimental setup displayed in panel b (left). ***P = 0.0001 (day 7), <0.0001 (days 6 and 8) (two-way ANOVA and Sidak’s multiple comparisons test; *n* = 4 and 3 mini-colons for calorie-restricted and -enriched diets, respectively). **e**, Brightfield images of mini-colons treated with the indicated diets according to experimental setup displayed in panel b (right). Images correspond to 6 days after tumorigenic induction. Scale bar, 75 μm. **f**, Multiplicity of tumours emerged in mini-colons treated with the indicated diets according to experimental setup displayed in panel b (right). Differences are not significant (two-way ANOVA and Sidak’s multiple comparisons test; *n* = 4 and 3 mini-colons for calorie-restricted and -enriched diets, respectively). **g**, Brightfield image of a healthy (non-transformed) mini-colon with integrated stromal cells in the extracellular matrix. Scale bar, 75 μm. **h**, Immunofluorescence image showing the presence of E-cadherin (green) and Vimentin (magenta) in the mini-colon shown in panel g. Scale bar, 75 μm. **i**, Brightfield images of mini-colons in the indicated mono- (left) and co-culture (right) setups. Images correspond to 6 days after tumorigenic induction. Scale bar, 75 μm. **j**, Brightfield image of an invasive front (arrowhead) formed in response to the presence of stromal cells in a mini-colon. The image corresponds to 6 days after tumorigenic induction (zoomed-in from panel i). Scale bar, 30 μm. **k**, Immunofluorescence image showing the presence of E-cadherin (green) and Vimentin (magenta) in the invasive front from panel j 23 days after tumorigenic induction. Scale bar, 20 μm. In **d** and **f**, data represent mean ± SEM. Source Data

Besides cell-intrinsic factors, colon tumorigenesis in vivo is heavily modulated by a myriad of environmental molecules that continuously contact the luminal side of colonocytes, such as the metabolites produced by colon-residing microbiota^29^. The impact of these molecules cannot be faithfully evaluated in conventional organoid cultures, as their lumen is not accessible. As mini-colons address this limitation, we also investigated whether they could model the role of bacterial metabolites of which the tumorigenic function has been corroborated in vivo. To that end, we administered specific metabolites exclusively in the luminal side of healthy mini-colons and, after a conditioning period of 2 days, induced oncogenic recombination (Fig. 4h). When luminally exposed to deoxycholic acid, a tumour-promoting metabolite^29–31^, mini-colons developed tumours with fast kinetics and high multiplicity (Fig. 4i,j). Conversely, both tumour-suppressive butyrate^29,32^ and β-hydroxybutyrate^33^ slowed tumour development and reduced multiplicity (Fig. 4i,j). These results demonstrate that mini-colons faithfully recapitulate the in vivo pathophysiological responses to bacterial metabolites, whereas conventional organoid cultures do not provide informative data on their tumorigenic relevance (Extended Data Fig. 10a).

Dietary components also constitute a relevant source of luminal factors conditioning colon tumorigenesis^34^. We therefore performed analogous experiments modelling diets with different caloric contents (Fig. 4h and Extended Data Fig. 10b). These revealed that calorie restriction in the luminal space effectively reduced tumour burden when compared to calorie-enriched medium (Extended Data Fig. 10c,d), consistent with in vivo evidence^35^. To show the relevance of luminal accessibility, we placed the same amount of dietary medium in the basal medium reservoirs instead of the luminal space (Extended Data Fig. 10b). Here, no differences were observed between the two dietary patterns (Extended Data Fig. 10e,f), therefore indicating that an accessible lumen—a forbidden feature in conventional organoids—is decisive for the physiologically relevant modelling of colon biology. Collectively, these findings demonstrate that the mini-colon is a versatile tool that enables faithful in vitro recapitulation of CRC tumorigenesis and its environmental determinants.

## Discussion

Here we show that the mini-colon model shifts the paradigm of cancer initiation research, allowing ex vivo tumorigenesis with unparalleled pathophysiological intricacy. Coupled with spatiotemporal control of oncogenesis, real-time single-cell resolution and broad experimental flexibility, this system opens new perspectives for in vitro screening of cellular and molecular determinants of cancer development. Supporting this, mini-colons faithfully reflect in vivo-like responses to microbiota-derived metabolites and dietary patterns. Likewise, our model can help in the discovery and validation of genetic targets and tumour-suppressive drugs, as illustrated by the finding that glutathione peroxidase inhibition abrogates CRC tumour development. This constitutes a major advance over conventional 3D culture systems like organoids and Transwell models, which can recapitulate isolated aspects of colon biology such as histopathological features^36^ or apical accessibility^37^, respectively, but lack the all-round topobiological complexity required to allow tumour formation ex vivo. Although such complexity demands bioengineering expertise to generate mini-colons, we have provided a detailed protocol that makes this system widely adoptable across laboratories that are already familiar with conventional organoid cultures (Protocol Exchange^38^; see Methods).

As for most genetic models of CRC, our system is based on the simultaneous acquisition of several mutations, which does not fully recapitulate the sequential tumorigenic process that occurs in vivo^39^. We therefore acknowledge that adopting a stepwise mutational system will enhance the relevance of the mini-colon as a cancer initiation model. We are also aware that spatial transcriptomics approaches will improve our understanding of tumour heterogeneity in the mini-colon. In the same lines, we envision the incorporation of additional regulatory layers in our OptoCre system, such as the fusion with the oestrogen receptor ligand-binding domain for subcellular localization control^40^, as a promising way to achieve finer spatiotemporal regulation of recombination.

Although mini-colons cannot be considered to be a general replacement for animals in all contexts of cancer research, they offer the possibility to reduce animal use in a wide range of experimental applications. Importantly, the pathophysiological relevance of the mini-colon can be readily enhanced by including stromal cells in the surrounding biomimetic extracellular matrix, which condition both tumour dynamics and invasiveness (Extended Data Fig. 10g–k). Current lines of work that will be made available in ensuing publications have also proved that this model can be applied to patient-derived colorectal cancer specimens. Lastly, we anticipate that, by adapting its biomechanical properties, topology and culture conditions, it will be possible to expand the system to other prominent epithelial cancer types, such as lung, breast or prostate, bringing an important experimental resource to multiple fields.

## Methods

### Mice

*Apc*^*fl/fl*^ mice (a gift from T. Petrova) were crossed to *Cdx2-cre*^*ERT2*^ mice (The Jackson Laboratory). *Apc*^*fl/fl*^*Cdx2-cre*^ERT2^ mice (termed A) were then crossed with *Kras*^*LSL-G12D/+*^*Trp53*^*fl/fl*^ mice (a gift from E. Meylan) to generate *Apc*^*fl/fl*^*Kras*^*LSL-G12D/+*^*Trp53*^*fl/fl*^*Cdx2-cre*^ERT2^ mice (termed AKP). AKP mice were then back-crossed with C57BL6/J (The Jackson Laboratory) to generate *Apc*^*fl/fl*^*Kras*^*LSL-G12D/+*^*Cdx2-cre*^*ERT2*^ mice (termed AK).

To induce tumorigenesis in vivo, Cre^ERT2^ recombinase was activated at the age of 8–10 weeks by a single intraperitoneal injection of 18 mg kg^–1^ tamoxifen (Sigma-Aldrich, T5648) in sunflower oil. Tumours were allowed to develop for 6 weeks. Mice were then sacrificed for tissue and cell isolation. See also below the specific section for transplantation of organoids in immunocompromised mice.

All animal work was conducted in accordance with Swiss national guidelines, reviewed and approved by the Service Veterinaire Cantonal of Etat de Vaud (VD3035.1 and VD3823). These regulations established 800 mm^3^ as the maximal subcutaneous tumour volume allowed, which was not exceeded in any of the experiments. In experiments in which tumorigenesis was induced in vivo, the locomotion, appearance, body condition and intestinal function of the mice were monitored twice weekly and assigned numerical scores to allow quantitative decision making in case humane end points were necessary before the predefined end point of the experiment (6 weeks). All of the mice in this study reached the predefined end point. Mice were kept in the animal facility under EPFL animal care regulations. They were housed in individual cages at 23 ± 1 °C and 55 ± 10% humidity under a 12 h–12 h light–dark cycle. All of the animals were supplied with food and water ad libitum.

### OptoCre module plasmid generation

The OptoCre module was designed by integrating the following constructs: (1) FUW-M2rtTA, which constitutively expresses the reverse tetracycline transactivator (rtTA); (2) FUW-tetO-GAVPO, which expresses the light-switchable trans-activator GAVPO after rtTA binding in the presence of doxycycline; and (3) FUW-OptoCre, which expresses Cre recombinase after GAVPO binding in the presence of blue light (Extended Data Fig. 1a). FUW-M2rtTA was purchased from Addgene (20342). Vectors containing GAVPO and the GAVPO-binding promoter (UASG)_5_-P_min_, developed previously^41^, were a gift from M. Thomson^42^. For FUW-tetO-GAVPO generation, GAVPO was subcloned into the doxycycline-responsive FUW-TetO backbone (Wernig Lab, Stanford) using the EcoRI and NheI restriction sites (Extended Data Fig. 1a). For FUW-OptoCre generation, (UASG)_5_-P_min_ was inserted into the FUW-TetO backbone from which the TetO promoter had been removed (Wernig Lab, Stanford) using the BstBI and BamHI restriction sites. We then introduced the Cre recombinase (Addgene, 25997) downstream of (UASG)_5_-P_min_ using the Pac1 restriction sites (Extended Data Fig. 1a).

### Isolation of colon cells

Healthy colon or tumour pieces were finely chopped using a scalpel and transferred to a gentle-MACS C-tube (Miltenyi, 130-093-237) containing 4 ml of digestion medium (RPMI (Thermo Fisher Scientific, 22400089), 1 mg ml^–1^ collagenase type IV (Life Technologies, 9001-12-1), 0.5 mg ml^–1^ dispase II (Life Technologies, 17105041) and 10 μg ml^–1^ DNase I (Applichem, A3778)). Tissues were then digested using the 37C_m_TDK_1 program on the gentle-MACS Octo Dissociator with heaters (Miltenyi). After the program was complete, the cell suspension was passed through a 70-μm strainer (Corning, 431751) and centrifugated at 400*g* for 5 min.

### Organoid and stromal cell culture

To establish organoids, colon cells were embedded in growth-factor-reduced Matrigel (Corning, 356231) (~2 × 10^4^ cells per 20 μl dome) and cultured in Advanced DMEM/F-12 (Thermo Fisher Scientific, 12634028) supplemented with 1× GlutaMax (Thermo Fisher Scientific, 35050038), 10 mM HEPES (Thermo Fisher Scientific, 15630056), 100 μg ml^−1^ penicillin–streptomycin (Thermo Fisher Scientific, 15140122), 1× B-27 supplement (Thermo Fisher Scientific, 17504001), 1× N2 supplement (Thermo Fisher Scientific, 17502001), 1 mM *N*-acetylcysteine (Sigma-Aldrich, A9165), 50 μg ml^−1^ primocin (InvivoGen, ant-pm-2), 50 ng ml^−1^ EGF (Peprotech, 315-09), 100 ng ml^−1^ noggin (produced at EPFL Protein Production and Structure Core Facility), 500 ng ml^−1^ R-spondin (produced at EPFL Protein Production and Structure Core Facility), 50 ng ml^–1^ WNT3A (Time Bioscience, rmW3aL-010), 10 mM nicotinamide (Calbiochem, 481907) and 2.5 μM Thiazovivin (Stemgen, AMS.04-0017). This full medium is termed ‘WENRNi’. The base version of this medium without EGF, noggin, R-spondin, WNT3A and nicotinamide is referred to as BMGF and was used for the expansion of colon tumour organoids since they do not require the additional growth factors. The base version of BMGF without B-27, N2 and *N*-acetylcysteine is termed BM or basal medium, and was used for growth-factor deprivation experiments. A detailed protocol describing organoid culture can be found elsewhere^9^. Where indicated, organoids were treated with the following compounds or growth factors: regorafenib (8 μM, Selleckchem, S1178), ripretinib (1 μM, Selleckchem, S8757), infigratinib (1 μM, Selleckchem, S2183), SCF (100 ng ml^–1^, PeproTech, 250-03), FGF2 (50 ng ml^–1^, Thermo Fisher Scientific, PMG0035) and IGF1 (100 ng ml^–1^, R&D Systems, 291-G1-200). Stromal cells were derived from cell suspensions from the primary tissue cultured in EGM-2 MV Microvascular Endothelial Cell Growth Medium-2 (Lonza, CC-3202) on conventional cell culture flasks. This medium selection strategy was followed by magnetic-activated cell sorting (MACS) on EPCAM (Miltenyi Biotec, 130-105-958) according to the manufacturer’s instructions to discard epithelial cells. The presence of stromal cells was further confirmed by immunofluorescence analyses of vimentin expression (see below). Cells were tested for mycoplasma before cryopreservation and in randomized routine checks using the MycoAlert PLUS Mycoplasma Detection Kit (Lonza, LT07-705).

### Generation of light-inducible cells

Lentiviral particles carrying the three components of the OptoCre module (see above; Extended Data Fig. 1b) and a Cre recombination reporter were produced at the EPFL Gene Therapy Platform by transfecting HEK293 cells with each plasmid of the OptoCre module and pLV-CMV-LoxP-DsRed-LoxP-eGFP (Addgene, 65726) plasmids. Lentivirus-containing supernatants were collected and concentrated by centrifugation (1,500*g* for 1 h at 4 °C). Lentiviral titration was performed using the p24-antigen ELISA (ZeptoMetrix, 0801111). For transduction, colon organoids (around 2 × 10^5^ cells) were dissociated into single cells by incubating in TrypLE Express Enzyme (Thermo Fisher Scientific, 12605028) at 37 °C for 5 min. Cells were then washed with basal medium supplemented with 10% fetal bovine serum (FBS) (Thermo Fisher Scientific, 10500064) and resuspended in WENRNi medium containing 8 μg ml^−1^ polybrene (Sigma-Aldrich, TR-1003-G) and the following amounts of viral particles: ~10 ng of p24 FUW-M2rtTA per ml, ~80 ng of p24 FUW-tetO-GAVPO per ml, ~80 ng of p24 FUW-OptoCre per ml and ~1,000 ng of p24 CMV-LoxP-DsRed-LoxP-eGFP per ml. These cells were plated in a 24-well plate, centrifuged at 600*g* for 60 min at room temperature, and incubated for 6 h at 37 °C. After incubation, the cells were collected, centrifuged, plated in 20 μl Matrigel domes in a 24-well plate and cultured in WENRNi medium. Cells expressing the Cre recombination reporter were selected by supplementing WENRNi medium with 8 μg ml^−1^ puromycin (InvivoGen, ant-pr-1).

### Light-mediated oncogenic recombination

The OptoCre module requires (1) doxycycline to induce rtTA-mediated GAVPO expression and (2) blue light to induce GAVPO-mediated Cre recombinase expression (Extended Data Fig. 1a,b). At the desired time of oncogenic induction, 2 μg ml^−1^ doxycycline hydrochloride (Sigma-Aldrich, D3072) was added to the culture medium of either the organoids or mini-colons. Light induction was then performed using a custom-made LightBox built by Baur SA and the Instant Lab at EPFL. The LightBox consisted of an Acqua A5 System (Acme Systems) that could be remotely parametrized using a custom-made web-based application. Communication between the Acqua A5 System and the microcontroller (PJRC, Teensy 3.2) was done through Blocky programming, which allowed for control of the LED drivers (Sparkfun, PicoDuck). The LEDs (Cree LEDs, XLamp XP-C Blue LEDs) were placed into a custom multilayer 24-well plate holder made of black anodized aluminium and polyphenylsulfone; the height was optimized for homogeneous light distribution within each well. The entire LightBox, plate-holder, LEDs and cables were made to be placed in the incubator (watertight and heat resistant). Diffusive elements (Luminit, Light Shaping Diffuser 80°) were used to render the illumination more homogeneous inside each well. The intensity of the blue light (450–465 nm, peak at 455 nm) was optimized, set to 100 μW cm^−2^ and shined on the cells for 3 h. After blue-light exposure, doxycycline was removed by washing the cultures with fresh medium. In experiments targeting the light to specific regions of the mini-colon, work was carried out in the dark using a near infrared light (Therabulb, NIR-A) to prevent leaky Cre expression. Light-targeting was performed using a photomask that was adapted to the dimensions of the mini-colon and that was created from a photoresist and chrome-coated standard 5 × 5 inch silica plate (Nanofilm) with an automated machine (VPG200 Heidelberg Instrument, 2.0 µm resolution). Once the exposed photoresist was developed, the chrome layer was wet-etched and the remaining photoresist was stripped using a mask processor (Hamatech HMR900)^9^.

### Microdevice design, fabrication and loading

The microfluidic device used for mini-colon cultures was designed using Clewin 3.1 (Phoenix Software) and fabricated as previously described^9^. It was composed of three main compartments: (1) a hydrogel chamber for cell growth in the centre; (2) two basal medium reservoirs flanking the hydrogel compartment; and (3) inlet and outlet channels for luminal perfusion^9^. An extracellular matrix containing 80% (v/v) type I collagen (5 mg ml^−1^, Reprocell, KKN-IAC-50) and 20% (v/v) growth–factor-reduced Matrigel was loaded into the hydrogel compartment. The microchannels constituting the mini-colon architecture within the hydrogel were designed using Adobe Illustrator CC 2019 and Wolfram Mathematica 11.3. They were then read by PALM RoboSoftware 4.6 (Zeiss) and ablated using a nanosecond laser system (1 ns pulses, 100 Hz frequency, 355 nm; PALM Micro-Beam laser microdissection system, Zeiss). The dimensions of the mini-colon architecture were described previously^9^. A detailed description of all the key steps required for the generation and maintenance of mini-guts is available at Protocol Exchange (10.21203/rs.3.pex-903/v1)^38^.

### Mini-colon culture, development and tumorigenesis

Colon organoids were dissociated into single cells by incubating in TrypLE Express Enzyme for 5 min at 37 °C followed by vigorous pipetting. This cell suspension was washed in 5 volumes of Advanced DMEM/F-12 supplemented with 10% FBS and passed through 40 μm cell strainers (Corning, 431750). After centrifugation at 400*g* for 5 min, cells were resuspended in WENRNi medium at around 10^6^ cells per ml. The mini-colon luminal microchannel was filled with 10 μl of this cell suspension. Cells were allowed to settle down in the mini-colon crypt-shaped cavities for 5 min, and the leftover unadhered cells were washed out from the microchannel by medium perfusion. The basal medium reservoirs were filled with 100 μl of WENRNi. Unless otherwise indicated, once the healthy colonic epithelium was fully formed (around 2 days after seeding), the medium in the luminal channel was switched to BM, while WENRNi was kept in the basal medium reservoirs. This gradient of growth factor from basal medium reservoirs to luminal space favours colonocyte differentiation across the crypt–lumen axis. For low-differentiation conditions of the differentiation experiments, WENRNi was kept in both the lumen and basal medium reservoirs. Conversely, high-differentiation mini-colons were cultured in WENRNi medium without WNT3A and nicotinamide (termed ENR). Unless otherwise stated, once the colonic epithelium was fully formed, oncogenic induction in the mini-colons was performed as stated above. Where indicated, tiopronin (5 mM, Selleckchem, S2062) or CHIR99021 (3 μM, StemCell Technologies, 100-1042) was added to the basal medium reservoirs after or before oncogenic induction, respectively. For co-culture experiments, ~500 stromal cells were seeded in each hydrogel before the laser-mediated ablation of the mini-colon pattern. The rest of the culture conditions and procedures remained unchanged. To avoid potential unspecific results derived from the small (but non-zero; Extended Data Fig. 1d,e) leakiness of the optogenetic system, each replication across all studies was performed using independent OptoCre organoid lines freshly generated before each experiment. In all cases, the mini-colons were incubated at 37 °C in 5% CO_2_ humidified air, with daily luminal perfusions and medium changes every other day.

### Mini-colon whole-mount immunofluorescence staining

Mini-colons were rinsed with phosphate-buffered saline (PBS) and fixed in 4% paraformaldehyde (Thermo Fisher Scientific, 15434389) overnight at 4 °C. After rinsing with PBS, the hydrogels were extracted from the PDMS scaffold using a scalpel, placed into a 48-well plate, permeabilized with 0.1% Tween-20 (Sigma-Aldrich, P9416) in PBS (10 min at 4 °C) and blocked in 2 mg ml^−1^ bovine serum albumin (Sigma-Aldrich, A3059) in PBS containing 0.1% Triton X-100 (Sigma-Aldrich, T8787) (blocking buffer) for at least 45 min at 4 °C. The samples were subsequently incubated overnight at 4 °C in blocking buffer with the corresponding following primary antibodies: CD44 (1:200; Abcam, ab157107), FABP1 (1:100; R&D Systems, AF1565), SOX9 (1:200; Abcam, ab185966), GPX2 (1:200; Bioss Antibodies, BS-13396R), IL-1α (1:200; R&D Systems, AF-400-SP), CDKN2A (1:100; Abcam, ab211542), E-cadherin (1:100; Abcam, ab11512) and vimentin (1:200; Abcam, ab92547). After three washes in blocking buffer for a total of 6 h at room temperature, the samples were incubated overnight at 4 °C in blocking buffer with the following corresponding secondary antibodies: Alexa Fluor 488 anti-goat (1:400, Thermo Fisher Scientific, A-11055), Alexa Fluor 488 anti-rat (1:400, Thermo Fisher Scientific, A-21208) and Alexa Fluor 647 anti-rabbit (1:400, Thermo Fisher Scientific, A-31573). After 3 washes in blocking buffer for a total of 6 h at room temperature, the samples were incubated with DAPI (1 μg ml^−1^; Tocris Bioscience, 5748) for 10 min at room temperature in blocking buffer. Before imaging, the hydrogels were mounted onto 35 mm glass bottom dishes (Ibidi, 81218-200) in Fluoromount-G (SouthernBiotech, 0100-01).

### Mini-colon sectioning and histochemistry

Mini-colons were fixed and extracted from the PDMS scaffold as indicated above and were prepared for cryosectioning by incubating in 30% (w/v) sucrose (Sigma-Aldrich, S1888) in PBS until the sample sank. Subsequently, the samples were incubated for 12 h in a mixture of Cryomatrix (Epredia, 6769006) and 30% sucrose (mixing ratio 50/50) followed by a 12 h incubation in pure Cryomatrix. The samples were then embedded in a tissue mould, frozen on dry ice, and cut into 40-µm-thick sections at −20 °C using the CM3050S cryostat (Leica). Haematoxylin and eosin staining was performed at the EPFL Histology Core Facility using the Ventana Discovery Ultra automated slide preparation system (Roche).

### Microscopy and image analysis

Bright-field and fluorescence imaging of living organoids and mini-colons was performed using the Nikon Eclipse Ti2 inverted microscope with ×4/0.13 NA, ×10/0.30 NA and ×40/0.3 NA air objectives and a DS-Qi2 camera (Nikon Corporation). Time lapses were taken in a Nikon Eclipse Ti inverted microscope system equipped with ×4/0.20 NA and ×10/0.30 NA air objectives and DS-Qi2 (Nikon Corporation) and Andor iXon Ultra 888 (Oxford Instruments) cameras. Both systems were controlled using the NIS-Elements AR software (Nikon Corporation). The extended depth of field (EDF) of bright-field images was calculated using a built-in NIS-Elements function. Fluorescence confocal imaging of fixed mini-colons was performed using the Leica SP8 STED 3X inverted microscope system equipped with ×10/0.30 NA air and ×25/0.95 NA water objectives, 405 nm diode and supercontinuum 470–670 nm lasers, and the system was controlled by the Leica LAS-X software (v.3.5.7, Leica microsystems). Histological sections were imaged using a Leica DM5500 upright microscope with ×10/0.30 NA and ×20/0.75 NA air objectives, a ×40/1.0 NA oil objective and a DMC 2900 Color camera, and the system was controlled by the Leica LAS-X software. Image processing was performed using standard contrast- and intensity-level adjustments in ImageJ (NIH). For oncogenic recombination analyses, the GFP-positive area was measured from 16-bit EDF images by subtracting the background, sharpening the images, and applying a signal threshold and a mask. The ratio between GFP-positive area and total organoid area was used for analyses. Recombined cells were segmented using StarDist with the default parameters (https://github.com/stardist) on the GFP channel of mini-colon images. Cell debris was discarded from segmentation analyses by setting an empirically established size threshold. For tumour quantification in the mini-colon, neoplastic structures with at least three times the thickness of the surrounding healthy epithelium were considered to be tumours. Videos of immunostainings were rendered using Imaris (Oxford Instruments).

### Mini-colon shedding evaluation

The medium from the luminal compartments of the mini-colons, together with an additional luminal perfusion of 10 μl of basal medium, was collected every day for 4 days after the blue-light-induced oncogenic recombination. The protein content in these extracts was analysed using conventional Bradford assays (Bio-Rad, 5000006) and used as an indicator of cell shedding.

### Mini-colon cell line derivation

Mini-colon-containing hydrogels were extracted from their microfluidic devices with a scalpel as indicated above and incubated with 0.1% (w/v) collagenase I (Thermo Fisher Scientific, 17100-017) at 37 °C for 10 min. Once the hydrogel was fully digested, the mini-colon was washed with PBS and digested with TrypLE Express Enzyme for 5 min at 37 °C. The resulting cell suspension was washed with Advanced DMEM/F-12 supplemented with 10% FBS, pelleted, embedded in Matrigel and cultured as indicated above for regular colon organoids.

### Transplantation of organoids in immunocompromised mice

Organoid lines were established as indicated above from either in vivo colon tumours (reference AKP) or tumour-bearing mini-colons (mini-colon AKP). These organoids were dissociated into single cells using TrypLE Express Enzyme for 5 min at 37 °C, washed with Advanced DMEM/F-12 supplemented with 10% FBS, pelleted and embedded in Matrigel at 2.5 × 10^6^ cells per ml. A total of 100 μl of this suspension was inoculated by subcutaneous injection into the right flank of NOD.*Cd-Prkdz*^*scid*^*Il2rg*^*tm1Wjl*^*/Szj* (NSG) mice (Jackson laboratories). Tumour growth was monitored using callipers twice per week until the end point at 18 days after inoculation. Length (*L*) and width (*W*) were measured and used to approximate the volume (*V*) of the tumour in mm^3^ using the modified ellipsoid formula: *V* = (*L* × *W*^2^)/2. After euthanasia, tumours were resected from the graft location and measured once more with callipers.

### Graft sectioning and histochemistry

Tumour samples were fixed overnight in 4% paraformaldehyde at 4 °C, dehydrated in graded ethanol baths, cleared with xylene, embedded in paraffin and cut into 4-µm-thick sections using the HM 325 Rotary Microtome (Thermo Fisher Scientific). These sections were mounted onto Superfrost plus slides (Epredia, J1800AMNZ) and allowed to dry for 2 days at room temperature. Haematoxylin and eosin staining was performed at the EPFL Histology Core Facility using the Ventana Discovery Ultra automated slide preparation system (Roche).

### Mutational screening in colon organoids

Genomic DNA was isolated from colon cells using the PureLink Genomic DNA Mini Kit (Thermo Fisher Scientific, K182001) according to the manufacturer’s instructions. Recombination of the LSL (LoxP-Stop-LoxP) cassette controlling *Kras*^*G12D*^ expression was confirmed by PCR using the protocol and oligos described by the Tyler Jacks laboratory (https://jacks-lab.mit.edu/, *Kras*^*G12D*^ Conditional PCR). *Apc* and *Trp53* recombinations were confirmed through exome sequencing performed at BGI Genomics at 100× coverage using DNBSEQ sequencing technology. DNA reads were mapped to the mouse GRCm39 genome assembly using BWA-MEM (v.0.7.17), filtered using samtools (v.1.9) and visualized using IGV (Integrative Genomics Viewer, Broad Institute, v.2.12.3).

### Organoid proliferation assays

Single-cell suspensions of colon cells were generated as indicated above and embedded in 10 μl Matrigel domes at around 10^4^ cells per dome in a 48-well plate. For each of the following 4 days, 220 μM resazurin (Sigma-Aldrich, R7017) was added to the culture medium and incubated for 4 h at 37 °C. Next, the resazurin-containing medium was collected and replaced with regular medium. Organoid proliferation was estimated by measuring the reduction of resazurin to fluorescent resorufin in the medium each day using the Tecan Infinite F500 microplate reader (Tecan) with 560 nm excitation and 590 nm emission filters. In the case of colony-formation assays, seeding was performed at around 10^3^ cells per dome and the resulting colonies were counted after 3 days.

### Organoid RNA extraction and bulk transcriptome profiling

Before RNA isolation, organoids were cultured for 3 days as indicated above and starved for 24 h in BM for the evaluation of growth-factor dependence. In the case of the *Gpx2-*knockdown experiments, 2 timepoints were analysed: 0 and 2 weeks after blue-light-induced activation (before and after oncogenic recombination, respectively). In all cases, cells were collected using TrypLE Express Enzyme as indicated above and lysed in RLT buffer (Qiagen, 74004), and the RNA was extracted using the QIAGEN RNeasy Micro Kit (Qiagen, 74004) according to the manufacturer’s instructions. Purified RNA was quality checked using a TapeStation 4200 (Agilent), and 500 ng was used for QuantSeq 3′ mRNA-seq library construction according to the manufacturer’s instructions (Lexogen, 015.96). Libraries were quality checked using a Fragment Analyzer (Agilent) and were sequenced in the NextSeq 500 (Illumina) system using NextSeq vm2.5 chemistry with Illumina protocol 15048776. Reads were aligned to the mouse genome (GRCm39) using star (v.2.7.0e)^43^. R (v.4.1.2) was used to perform the differential expression analyses. Count values were imported and processed using edgeR^44^. Expression values were normalized using the trimmed mean of *M* values (TMM) method^45^ and low-expressed genes (<1 counts per million) were filtered out. Differentially expressed genes were identified using linear models (Limma-Voom)^46^ and *P* values were adjusted for multiple comparisons using the Benjamini–Hochberg correction method^47^. Volcano plots and heat maps were generated using the EnhancedVolcano (https://github.com/kevinblighe/EnhancedVolcano) and heatmap3 (https://github.com/slzhao/heatmap3) packages, respectively. The in vivo AKP signature was established from the differentially expressed genes between in vivo and organoid AKP lines with a log_2_-transformed fold change of at least |2|. To evaluate the enrichment of the in vivo AKP gene expression program across samples, the enrichment scores for both the upregulated and downregulated signatures were calculated using single-sample GSEA (ssGSEA)^48^. The difference between the two normalized enrichment scores yielded the fit score. ssGSEA was also used to analyse the enrichment of the MSigDB curated Hallmark gene set^49^ in *Gpx2*-knockdown organoids. Functional annotation was performed using DAVID^50^ on the genes with a log_2_-transformed fold change of at least |1|. GOplot^51^ was used for the integration of expression and functional annotation data. Known functional interactions among relevant genes were obtained through STRING^52^. Cytoscape^53^ was used to perform network data integration and visualization.

### Single-cell transcriptome profiling and lineage tracing

Lineage tracing was performed using the CellTag system^22^ (V1 pooled barcode library, Addgene, 115643-LVC). In brief, we co-transduced inducible colon organoids with the CellTag barcode library (multiplicity of infection of around 5) and the OptoCre module as indicated above. These cells were then introduced and induced in the mini-colon system as indicated before. After 7 days in the system and when mini-colon tumours were clearly visible, we extracted the cells from mini-colons as indicated above. After pooling and filtering (40 μm) the cell suspensions from two mini-colons, the single-cell sequencing library was constructed using 10x Genomics Chromium 3′ reagents v3.1 according to the manufacturer’s instructions (10x Genomics, PN-1000269, PN-1000127, PN-1000215). Sequencing was performed using NovaSeq 6000 v1.5 reagents (Illumina protocol #1000000106351 v03) for around 100,000 reads per cell. The reads were aligned using Cell Ranger (v.6.1.2)^54^ to the mouse genome (mm10) carrying artificial chromosomes for both GFP and CellTag UTR genes, as recommended by CellTag developers for facilitating barcode identification^55^. Raw count matrices were imported into R and analysed using Seurat (v.4.2.0)^56^. Dead cells were discarded on the basis of the number of detected genes (less than 3,000) and the percentage of mitochondrial genes (more than 20%), leading to 2,429 cells after filtering. The data were log-normalized and scaled, and dimensionality reduction was conducted using UMAP with 10 dimensions. Louvain clustering yielded 17 clusters that were merged and named on the basis of canonical cell type markers. Stem, cycling, progenitor, goblet and enteroendocrine cell scoring was based on published signatures in mini-intestines and in vivo^9^. Gene sets highlighting bottom, middle and top colonocytes were taken from enterocyte zonation studies^23^. Cancer stemness was scored based on the expression of *Lgr5*, *Cd44* and *Sox9*. Intrinsic consensus molecular subtype (iCMS) signatures for colorectal cancer were obtained from published work^27^. Signature scoring was performed using burgertools (https://github.com/nbroguiere/burgertools). Visual representations of the data were generated using Seurat internal functions. For lineage-tracing analyses, CellTag detection, quantification and clone calling were performed as indicated by CellTag developers^55^, excluding cells expressing fewer than 2 or more than 30 CellTags. After filtering, 83 clonal populations were identified, from which only those with a minimum size of 5 cells were considered for further analyses. To identify clonal populations belonging to tumour cells, we looked for cells expressing transcripts carrying the genetically engineered *Apc* and *Trp53* mutations, that is, deletions of exons 15 and 2–10, respectively (Extended Data Figs. 3g and 6b,c). Note that this approach could not be performed for *Kras*, as the mutation is also present in the transcripts from WT cells (but not expressed). As scRNA-seq provides low coverage on exon junctions and therefore the presence of mutations can be assessed only in a small fraction of cells, we used both the cell-type composition and size distributions of bona fide mutationally confirmed tumour clonal populations to classify the rest of clones. Those falling within plus or minus 2 s.d. of the mean cell composition and size of bona fide tumours were classified as tumour clonal populations. Healthy clones were defined as those with a clearly distinct (outside the aforementioned range) cell type composition and the same upper limit size as was observed for tumour clones. After filtering and classification, 16 healthy and 18 tumour clonal populations were obtained and used for further analyses (Extended Data Fig. 6d). To define the most robust tumour-clone-specific markers, the gene expression from cells in each clone was compared to that from cells in each other clone using the Wilcoxon rank-sum test. We considered only the positive markers and selected those with adjusted *P* < 10^−5^. The association of these markers with clinical parameters in patients with CRC (survival, lymph node staging) was performed through cBioPortal (https://www.cbioportal.org/) using the 640-sample CRC TCGA dataset (https://www.cancer.gov/tcga) and a differential expression threshold equal or greater than |2 |. Further information is provided in the Data availability and Code availability sections.

### shRNA-mediated transcript knockdown

Organoids were transduced as indicated above with lentiviral particles encoding *Gpx2* shRNAs obtained from Sigma-Aldrich (TRCN0000076529, TRCN0000076531 and TRCN0000076532; sh*Gpx2* 1, sh*Gpx2* 2 and sh*Gpx2* 3, respectively) or, as a control, shRNA-free counterparts (Addgene, 65726). Transduced cells were selected with puromycin (5 μg ml^−1^; InvivoGen, ant-pr-1). Proper transcript knockdown was assessed using quantitative PCR with reverse transcription (RT–qPCR) and RNA-seq.

### Analysis of mRNA abundance

Organoids were cultured and collected as indicated above. Cells were then lysed in RLT buffer and RNA was extracted using the QIAGEN RNeasy Micro Kit as indicated above. RT–qPCR was performed using the iTaq Universal SYBR Green One-Step Kit (Bio-Rad Laboratories, 1725150) and the QuantStudio 7 Flex Real-Time PCR System (Thermo Fisher Scientific, 4485701). Raw data were analysed using Design & Analysis Software (v.2.6.0, Thermo Fisher Scientific). We used the abundance of the endogenous *Gapdh* mRNA as internal normalization control. The following primers were used for transcript quantification: 5′-AGTTCGGACATCAGGAGAACTG-3′ (forward, *Gpx2*), 5′-GATGCTCGTTCTGCCCATTG-3′ (reverse, *Gpx2*), 5′-ATCCTGCACCACCAACTGCT-3′ (forward, *Gapdh*) and 5′-GGGCCATCCACAGTCTTCTG-3′ (reverse, *Gapdh*).

### Microbiota and diet modelling

Inducible mini-colons were generated as indicated above. Once the epithelium was formed and before oncogenic induction, mini-colons were subjected to a conditioning period of 2 days in which luminal medium was (1) supplemented with 100 μM deoxycholate (Sigma-Aldrich, D2510), 10 mM butyrate (Sigma-Aldrich, B5887) or 10 mM β-hydroxybutyrate (Sigma-Aldrich, 54965); or (2) replaced with MEMα (calorie-restricted condition, Thermo Fisher Scientific, 22561-021) or Advanced DMEM/F12 supplemented with 30 μM palmitic acid (calorie-enriched condition, Sigma-Aldrich, P0500). The same concentrations were used in organoid control experiments, but these were added to the full culture medium as the luminal compartment is not accessible in organoids. To assess the relevance of luminal exposure to these factors in the mini-colon, the same total amounts were added in the basal medium reservoirs instead of the luminal channel. In all cases, after conditioning, oncogenic recombination was performed and the mini-colon was cultured as indicated above. The different medium compositions were replenished every day during luminal perfusion.

### Statistics and reproducibility

The number of biological replicates (*n*), the type of statistical tests performed and the statistical significance for each experiment are indicated in the corresponding figure legend. For images associated with quantification charts (Fig. 1b,c with Fig. 1e; Fig. 2b with Fig. 2c; Fig. 2d with Extended Data Fig. 4b; Fig. 2f with Fig. 2g; Fig. 4b with Extended Data Fig. 9a; Fig. 4i with Fig. 4j; Extended Data Fig. 2a with Fig. 1e; Extended Data Fig. 3d,e with Extended Data Fig. 3b; Extended Data Fig. 3h with Fig. 1e; Extended Data Fig. 5b with Extended Data Fig. 5c; Extended Data Fig. 5d with Extended Data Fig. 5e; Extended Data Fig. 9b with Extended Data Fig. 9c; Extended Data Fig. 9e with Extended Data Fig. 9f; Extended Data Fig. 9g with Fig. 4c; Extended Data Fig. 9l with Extended Data Fig. 9m; Extended Data Fig. 9n with Fig. 4g; Extended Data Fig. 10c with Extended Data Fig. 10d; Extended Data Fig. 10e with Extended Data Fig. 10f), the number of replicates is the same as for the corresponding chart and is indicated in the figure legend of the latter. For the rest of representative images (Figs. 1d and 3h and Extended Data Figs. 1f, 2b,c,f, 3a, 4a, 7d, 9h and 10a,g–k), three independent experiments were performed. scRNA-seq (Fig. 3a) and exome sequencing with matched PCR (Extended Data Fig. 3f,g) were performed with two independent sets of samples. Bulk RNA-seq was performed with at least three independent sets of samples. Unless otherwise indicated, statistical analyses were performed using GraphPad Prism v.9 (GraphPad). Data normality and equality of variances were analysed with Shapiro–Wilk and Bartlett’s tests, respectively. Parametric distributions were analysed using the Student’s *t*-test (when comparing two experimental groups) or ANOVA followed by either Dunnett’s test (when comparing more than two experimental groups with a single control group) or Tukey’s HSD test (when comparing more than two experimental groups with every other group). Nonparametric distributions were analysed using either Mann–Whitney *U*-tests (for comparisons of two experimental groups) or the Kruskal–Wallis followed by Dunn’s test (for comparisons of three or more than three experimental groups) tests. Sidak’s multiple-comparison test was used when comparing different sets of means. *χ*^2^ tests were used to determine the significance of the differences between expected and observed frequencies. In all cases, values were considered to be significant when *P* ≤ 0.05. Data obtained are given as the mean ± s.e.m.

### Reporting summary

Further information on research design is available in the Nature Portfolio Reporting Summary linked to this article.

## Online content

Any methods, additional references, Nature Portfolio reporting summaries, source data, extended data, supplementary information, acknowledgements, peer review information; details of author contributions and competing interests; and statements of data and code availability are available at 10.1038/s41586-024-07330-2.

## Supplementary information

Reporting Summary

Supplementary Table 1 Differentially expressed genes in shGpx2 colon organoids before oncogenic recombination.

Supplementary Table 2 Differentially expressed genes in shGpx2 colon organoids after oncogenic recombination

Supplementary Video 1 Early response to oncogenic activation within a mini-colon. 46 h time-lapse video of mutated cells in a mini-colon 24 h after oncogenic recombination.

Supplementary Video 2 Hyperplasia and early tumour development in a mini-colon. 36 h time-lapse video of a mini-colon with multiple tumour-initiating events 5 days after oncogenic recombination.

Supplementary Video 3 Ex vivo tumour development in a mini-colon. 38 h time-lapse video of tumour development in a mini-colon 9 days after oncogenic recombination.

Supplementary Video 4 Cancer stem cells initiate tumour development in mini-colons. 3D visualization of cancer stem cell marker CD44 overexpression in early tumorigenic sites.

Supplementary Video 5 Intratumour complexity in mini-colons. 3D visualization of CD44 (cancer stem cell marker) and FABP1 (mature colonocyte marker) expression in mini-colon tumours and epithelium.

## Source data

Source Data Fig. 1

Source Data Fig. 2

Source Data Fig. 3

Source Data Fig. 4

Source Data Extended Data Fig. 1

Source Data Extended Data Fig. 2

Source Data Extended Data Fig. 3

Source Data Extended Data Fig. 4

Source Data Extended Data Fig. 5

Source Data Extended Data Fig. 8

Source Data Extended Data Fig. 9

Source Data Extended Data Fig. 10

## Data Availability

Bulk and single-cell RNA-seq data reported in this paper have been deposited at the Gene Expression Omnibus (GEO) public repository under accession number GSE221163. The association analysis with clinical parameters in patients with CRC was performed through cBioPortal (https://cbioportal.org) using the 640-sample CRC TCGA dataset (https://cancer.gov/tcga). Source data are provided with this paper.

## Code availability

The code used for data analysis is available at GitHub (https://github.com/LorenzoLF/Mini-colon_bioengineering)^57^ and Zenodo (10.5281/zenodo.10057882)^58^.
