# Supplementary material for: Spatiotemporally resolved colorectal oncogenesis in mini-colons ex vivo
Source: Nature. 2024 Apr 24;629(8011):450–7. doi: 10.1038/s41586-024-07330-2 (PMC11078756; doi:10.1038/s41586-024-07330-2)
Supplement: Supplementary file 1 — Reporting Summary [file 41586_2024_7330_MOESM1_ESM.pdf]

nature portfolio

Corresponding author(s): Matthias P. Lutolf, L. Francisco Lorenzo-Martín

Last updated by author(s): Feb 28, 2024

Reporting Summary

Nature Portfolio wishes to improve the reproducibility of the work that we publish. This form provides structure for consistency and transparency in reporting. For further information on Nature Portfolio policies, see our Editorial Policies and the Editorial Policy Checklist.

Statistics

For all statistical analyses, confirm that the following items are present in the figure legend, table legend, main text, or Methods section.

n/a	Confirmed	
☐	☒	The exact sample size (*n*) for each experimental group/condition, given as a discrete number and unit of measurement
☐	☒	A statement on whether measurements were taken from distinct samples or whether the same sample was measured repeatedly
☐	☒	The statistical test(s) used AND whether they are one- or two-sided<br>*Only common tests should be described solely by name; describe more complex techniques in the Methods section.*
☐	☒	A description of all covariates tested
☐	☒	A description of any assumptions or corrections, such as tests of normality and adjustment for multiple comparisons
☐	☒	A full description of the statistical parameters including central tendency (e.g. means) or other basic estimates (e.g. regression coefficient) AND variation (e.g. standard deviation) or associated estimates of uncertainty (e.g. confidence intervals)
☐	☒	For null hypothesis testing, the test statistic (e.g. *F*, *t*, *r*) with confidence intervals, effect sizes, degrees of freedom and *P* value noted<br>*Give P values as exact values whenever suitable.*
☒	☐	For Bayesian analysis, information on the choice of priors and Markov chain Monte Carlo settings
☐	☒	For hierarchical and complex designs, identification of the appropriate level for tests and full reporting of outcomes
☐	☒	Estimates of effect sizes (e.g. Cohen's *d*, Pearson's *r*), indicating how they were calculated

Our web collection on statistics for biologists contains articles on many of the points above.

Software and code

Policy information about availability of computer code

Data collection	NIS-Elements AR 5.11.02 (Nikon Corporation), LAS-X software 3.5.7 (Leica), NanoDrop 2000/2000c (Thermo Scientific), QuantStudio version 7 Flex Real-Time PCR System, 10x Genomics Chromium and Illumina sequencing.

Data analysis	Clewin 3.1 (Phoenix Software) was used to produce the design for the photolitographic masks used to fabricate the SU8 molds of the devices. Adobe Illustrator CC 2019 and Wolfram Mathematica 11.3 were used to design micro-track coordinates, readable by PALM RoboSoftware 4.6 (Zeiss). Image processing was performed using standard contrast- and intensity-level adjustments in ImageJ version 2.7.0 (NIH). Recombined cells were segmented using StarDist version 0.8.3. Videos of immunostainings were rendered using Imaris version 9.9 (Oxford Instruments). DNA reads were mapped to the mouse GRCm39 genome assembly using BWA-MEM (v0.7.17), filtered using samtools (v1.9) and visualized using IGV (Integrative Genomics Viewer, Broad Institute, v2.12.3). RNA reads were aligned to the mouse genome (GRCm39) using star (version 2.7.0e). R (v4.1.2) was used to perform the differential expression analyses. Count values were imported and processed using edgeR (v3.36.0). Differentially expressed genes were identified using linear models (Limma-Voom) (v3.50.1). Volcano plots and heatmaps were generated using EnhancedVolcano (v1.19.0) and heatmap3 (v1.1.9) packages, respectively. To evaluate the enrichment of gene expression program across samples, the enrichment scores for both the upregulated and downregulated signatures were calculated using single-sample GSEA (v10.0.1). Functional annotation was performed using DAVID (version 2021). GOplot (v1.0.2) was used for the integration of expression and functional annotation data. Known functional interactions among relevant genes were obtained through STRING (v11.5). Cytoscape (v3.7.2) was used to perform network data integration and visualization. Single-cell RNAseq reads were aligned using Cell Ranger v6.1.2. Raw count matrices were imported into R and analyzed using Seurat v4.2.0. Single-cell signature scoring was carried out using burgertools (version 0.0.0.9000). Raw qRT-PCR data were analyzed using the Design & Analysis Software software (v2.6.0, Thermo Fisher Scientific). Statistical analyses were performed using GraphPad Prism version 9 (GraphPad Software). The code used for transcriptomic data analysis is available in GitHub at https://github.com/LorenzoLF/Mini-colon_bioengineering and in Zenodo under the DOI 10.5281/zenodo.10057882 (https://doi.org/10.5281/zenodo.10057882).

For manuscripts utilizing custom algorithms or software that are central to the research but not yet described in published literature, software must be made available to editors and reviewers. We strongly encourage code deposition in a community repository (e.g. GitHub). See the Nature Portfolio guidelines for submitting code & software for further information.

Data

Policy information about availability of data

All manuscripts must include a data availability statement. This statement should provide the following information, where applicable:

- Accession codes, unique identifiers, or web links for publicly available datasets
- A description of any restrictions on data availability
- For clinical datasets or third party data, please ensure that the statement adheres to our policy

Bulk and single-cell RNAseq data reported in this paper have been deposited in the Gene Expression Omnibus (GEO, ncbi.nlm.nih.gov/geo) public repository under the accession number GSE221163. The association analysis with clinical parameters in CRC patients were carried out through cBioPortal (cbioportal.org) using the 640-sample CRC TCGA dataset (cancer.gov/tcga).

Human research participants

Policy information about studies involving human research participants and Sex and Gender in Research.

Reporting on sex and gender	Not applicable.
Population characteristics	Not applicable.
Recruitment	Not applicable.
Ethics oversight	Not applicable.

Note that full information on the approval of the study protocol must also be provided in the manuscript.

Field-specific reporting

Please select the one below that is the best fit for your research. If you are not sure, read the appropriate sections before making your selection.

☒ Life sciences ☐ Behavioural & social sciences ☐ Ecological, evolutionary & environmental sciences

For a reference copy of the document with all sections, see nature.com/documents/nr-reporting-summary-flat.pdf

Life sciences study design

All studies must disclose on these points even when the disclosure is negative.

Sample size	The study is aimed at the development of a novel bioengineering concept. No specific sample size calculation was performed. Sample size was selected based on the observed variance in mini-colon or organoid development and responses. At least three independent replicates were performed in all experiments involving hypothesis testing. The exact sample size used for each experiment is indicated at the corresponding figure legend and in Methods.
Data exclusions	No data were excluded.

Replication	At least three independent replicates were performed in all experiments involving hypothesis testing. The exact sample size used for each experiment is indicated at the corresponding figure legend and in Methods. Source Data files are also provided.
Randomization	Mini-colons, organoids, and animals were randomly allocated into the experimental groups.
Blinding	The investigators were blinded to group allocation during sequencing and imaging data analysis. For other assays, investigators were not blinded with respect to the identities of the samples as it was required for proper experimental execution (for example, long-term mini-colon treatments). Even in these cases, the investigators were blinded during data quantitation and analysis.

Reporting for specific materials, systems and methods

We require information from authors about some types of materials, experimental systems and methods used in many studies. Here, indicate whether each material, system or method listed is relevant to your study. If you are not sure if a list item applies to your research, read the appropriate section before selecting a response.

Materials & experimental systems

n/a	Involved in the study
☐	☒ Antibodies
☐	☒ Eukaryotic cell lines
☒	☐ Palaeontology and archaeology
☐	☒ Animals and other organisms
☒	☐ Clinical data
☒	☐ Dual use research of concern

Methods

n/a	Involved in the study
☒	☐ ChIP-seq
☒	☐ Flow cytometry
☒	☐ MRI-based neuroimaging

Antibodies

Antibodies used	CD44 (1:200; Abcam, Catalog No. ab157107), Fabp1 (1:100; R&D systems, Catalog No. AF1565), Sox9 (1:200; Abcam, Catalog No. ab185966), Gpx2 (1:200; Bioss Antibodies, Catalog No. BS-13396R), IL-1 alpha (1:200; R&D Systems, Catalog No. AF-400-SP), Cdkn2a (1:100; Abcam, Catalog No. ab211542), E-cadherin (1:100; Abcam, Catalog No. ab11512), Vimentin (1:200; Abcam, Catalog No. ab92547), Alexa Fluor 488 anti-goat (1:400, Thermo Fisher Scientific, Catalog No. A-11055), Alexa Fluor 488 anti-rat (1:400, Thermo Fisher Scientific, Catalog No. A-21208), and Alexa Fluor 647 anti-rabbit (1:400, Thermo Fisher Scientific, Catalog No. A-31573).
Validation	Commercially-available antibodies (see above) have been validated by the respective manufacturers for the application and species they have been used for in our study. This information is available at each manufacturer's website:<br>CD44 (Abcam, Catalog No. ab157107): https://www.abcam.com/products/primary-antibodies/cd44-antibody-ab157107.html<br>Fabp1 (R&D systems, Catalog No. AF1565): https://www.rndsystems.com/products/human-mouse-rat-fabp1-l-fabp-antibody_af1565<br>Sox9 (Abcam, Catalog No. ab185966): https://www.abcam.com/products/primary-antibodies/sox9-antibody-epr14335-78-ab185966.html<br>Gpx2 (Bioss Antibodies, Catalog No. BS-13396R): https://www.biossusa.com/products/bs-13396r<br>IL-1 alpha (R&D Systems, Catalog No. AF-400-SP): https://www.rndsystems.com/products/mouse-il-1alpha-il-1f1-antibody_af-400-na<br>Cdkn2a (Abcam, Catalog No. ab211542): https://www.abcam.com/products/primary-antibodies/cdkn2ap16ink4a-antibody-epr20418-ab211542.html<br>E-cadherin (Abcam, Catalog No. ab11512): https://www.abcam.com/products/primary-antibodies/e-cadherin-antibody-decma-1-intercellular-junction-marker-ab11512.html<br>Vimentin (Abcam, Catalog No. ab92547): https://www.abcam.com/products/primary-antibodies/vimentin-antibody-epr3776-cytoskeleton-marker-ab92547.html<br>Alexa Fluor 488 anti-goat (Thermo Fisher Scientific, Catalog No. A-11055): https://www.thermofisher.com/antibody/product/Donkey-anti-Goat-IgG-H-L-Cross-Adsorbed-Secondary-Antibody-Polyclonal/A-11055<br>Alexa Fluor 488 anti-rat (Thermo Fisher Scientific, Catalog No. A-21208): https://www.thermofisher.com/antibody/product/Donkey-anti-Rat-IgG-H-L-Highly-Cross-Adsorbed-Secondary-Antibody-Polyclonal/A-21208<br>Alexa Fluor 647 anti-rabbit (Thermo Fisher Scientific, Catalog No. A-31573): https://www.thermofisher.com/antibody/product/Donkey-anti-Rabbit-IgG-H-L-Highly-Cross-Adsorbed-Secondary-Antibody-Polyclonal/A-31573

Eukaryotic cell lines

Policy information about cell lines and Sex and Gender in Research

Cell line source(s)	Primary colon organoid cell lines were established from Apcfl/fl Cdx2-CreERT2 (A), Apcfl/fl;KrasLSL-G12D/+ Cdx2-CreERT2 (AK) and Apcfl/fl;KrasLSL-G12D/+;Trp53fl/fl Cdx2-CreERT2 (AKP) mice. HEK293 cells were provided and used by the EPFL Gene Therapy Platform.
Authentication	Cell line authentication (genotyping) was performed through PCR and/or DNA sequencing.
Mycoplasma contamination	All cell lines tested negative for mycoplasma contamination.
Commonly misidentified lines (See ICLAC register)	No commonly misidentified cell lines were used for experiments in this study.

Animals and other research organisms

Policy information about studies involving animals; ARRIVE guidelines recommended for reporting animal research, and Sex and Gender in Research

Laboratory animals	Species: Mus musculus (mice). Apcfl/fl mice (a kind gift from Tatiana Petrova, University of Lausanne, Switzerland) were crossed to Cdx2-CreERT2 mice (The Jackson Laboratory, Charles River, L'Arbresle, France). Apcfl/fl Cdx2-CreERT2 mice (termed "A") were then crossed with KrasLSL-G12D/+;Trp53fl/fl mice (a kind gift from Etienne Meylan, Ecole Polytechnique Fédérale de Lausanne, Switzerland) to generate Apcfl/fl;KrasLSL-G12D/+;Trp53fl/fl Cdx2-CreERT2 mice (termed "AKP"). AKP mice were then back-crossed with C57BL6/J (The Jackson Laboratory, Charles River, L'Arbresle, France) to generate Apcfl/fl;KrasLSL-G12D/+ Cdx2-CreERT2 mice (termed "AK"). All animal work was conducted at an age of 8–10 weeks, in accordance with Swiss national guidelines, reviewed and approved by the Service Veterinaire Cantonal of Etat de Vaud, license numbers VD3035.1 and VD3823. Mice were kept in the animal facility under EPFL animal care regulations. They were housed in individual cages at 23°C +/- 1°C and 55% +/- 10% humidity with a 12-h light/dark cycle. All animals were supplied with food and water ad libitum.
Wild animals	The study did not involve wild animals.
Reporting on sex	The study is aimed at the development of a novel bioengineering concept. Sex was not considered in the study design.
Field-collected samples	The study did not involve samples collected from the field.
Ethics oversight	All animal work was conducted in accordance with Swiss national guidelines, reviewed and approved by the Service Veterinaire Cantonal of Etat de Vaud, license numbers VD3035.1 and VD3823. Mice were kept in the animal facility under EPFL animal care regulations.

Note that full information on the approval of the study protocol must also be provided in the manuscript.
